# An integrative drug repositioning framework discovered a potential therapeutic agent targeting COVID-19

**DOI:** 10.1038/s41392-021-00568-6

**Published:** 2021-04-24

**Authors:** Yiyue Ge, Tingzhong Tian, Suling Huang, Fangping Wan, Jingxin Li, Shuya Li, Xiaoting Wang, Hui Yang, Lixiang Hong, Nian Wu, Enming Yuan, Yunan Luo, Lili Cheng, Chengliang Hu, Yipin Lei, Hantao Shu, Xiaolong Feng, Ziyuan Jiang, Yunfu Wu, Ying Chi, Xiling Guo, Lunbiao Cui, Liang Xiao, Zeng Li, Chunhao Yang, Zehong Miao, Ligong Chen, Haitao Li, Hainian Zeng, Dan Zhao, Fengcai Zhu, Xiaokun Shen, Jianyang Zeng

**Affiliations:** 1 Institute for Interdisciplinary Information Sciences, Tsinghua University, Beijing, China; 2 NHC Key laboratory of Enteric Pathogenic Microbiology, Jiangsu Provincial Center for Diseases Control and Prevention, Nanjing, Jiangsu Province China; 3 Shanghai Institute of Materia Medica, Chinese Academy of Sciences, Shanghai, China; 4 Silexon AI Technology Co., Ltd., Nanjing, Jiangsu Province China; 5 Department of Computer Science, University of Illinois at Urbana-Champaign, Illinois, IL USA; 6 School of Pharmaceutical Sciences, Tsinghua University, Beijing, China; 7 School of Electronic Information and Communications, Huazhong University of Science and Technology, Wuhan, Hubei Province China; 8 Institute of Pathology, Tongji Hospital, Tongji Medical College, Huazhong University of Science and Technology, Wuhan, Hubei Province China; 9 Department of Automation, Tsinghua University, Beijing, China; 10 Inner Mongolia Alashan League Organization Establishment Committee Office Electronic Support Center, Alashan, Inner Mongolia China; 11 Convalife (Shanghai) Co., Ltd., Shanghai, China; 12 Advanced Innovation Center for Human Brain Protection, Beijing Tiantan Hospital, Capital Medical University, Beijing, China; 13 Beijing Advanced Innovation Center for Structural Biology, Tsinghua-Peking Joint Center for Life Sciences, Department of Basic Medical Sciences, School of Medicine, Tsinghua University, Beijing, China; 14 Center for Global Health, Nanjing Medical University, Nanjing, Jiangsu Province China

**Keywords:** Drug screening, Respiratory tract diseases, Infectious diseases

## Abstract

The global spread of severe acute respiratory syndrome coronavirus 2 (SARS-CoV-2) requires an urgent need to find effective therapeutics for the treatment of coronavirus disease 2019 (COVID-19). In this study, we developed an integrative drug repositioning framework, which fully takes advantage of machine learning and statistical analysis approaches to systematically integrate and mine large-scale knowledge graph, literature and transcriptome data to discover the potential drug candidates against SARS-CoV-2. Our in silico screening followed by wet-lab validation indicated that a poly-ADP-ribose polymerase 1 (PARP1) inhibitor, CVL218, currently in Phase I clinical trial, may be repurposed to treat COVID-19. Our in vitro assays revealed that CVL218 can exhibit effective inhibitory activity against SARS-CoV-2 replication without obvious cytopathic effect. In addition, we showed that CVL218 can interact with the nucleocapsid (N) protein of SARS-CoV-2 and is able to suppress the LPS-induced production of several inflammatory cytokines that are highly relevant to the prevention of immunopathology induced by SARS-CoV-2 infection.

## Introduction

The global COVID-19 pandemic caused by the novel coronavirus SARS-CoV-2 (2019-nCoV) has brought a huge number of infections and deaths worldwide according to the World Health Organization. More than 200 countries or regions around the world have reported to have confirmed COVID-19 cases and the number is still in a rapid increase, indicating that this novel coronavirus has posed a severe global health threat. Under the current circumstance of the absence of the specific vaccines and medicines targeting SARS-CoV-2, it is urgent to discover effective therapies especially drugs to treat the resulting COVID-19 disease and prevent the virus from further spreading. Considering that the development of a new drug generally takes years, probably the best therapeutic shortcut is to apply the drug repositioning strategy (i.e., finding the new uses of old drugs)^1–3^ to identify the potential antiviral effects against SARS-CoV-2 of existing drugs that have been approved for clinical use or to enter clinical trials. Those existing drugs with potent antiviral efficacy can be directly applied to treat COVID-19 in a short time, as their safety has been verified in principle in clinical trials.

In this study, we applied an integrative framework that fully takes advantage of machine learning and statistical analysis methods to systematically integrate large-scale available coronavirus-related data and identify the drug candidates against SARS-CoV-2 from a set of over 6000 drug candidates (mainly including approved, investigational, and experimental drugs). Our in silico screening process followed by experimental validation revealed that a poly-ADP-ribose polymerase 1 (PARP1) inhibitor, CVL218, currently in Phase I clinical trial, may serve as a potential drug candidate to treat COVID-19. Our in vitro assays demonstrated that CVL218 can exhibit effective inhibitory activity against SARS-CoV-2 replication in a dose-dependent manner and with no obvious cytopathic effect, and the efficacy of CVL218 can be further enhanced by a drug combination with another anti-SARS-CoV-2 drug candidate favipiravir. In addition, our surface plasmon resonance (SPR) binding assay indicated that CVL218 can interact with the nucleocapsid (N) protein of SARS-CoV-2 with a high affinity. Moreover, we found that in human peripheral blood mononuclear cells (PBMCs), CVL218 is able to suppress the LPS-induced production of several inflammatory cytokines, which have been reported previously to be of high relevance to the viral pathogenesis of COVID-19, especially for those intensive care unit (ICU) patients infected by SARS-CoV-2. Based on the data present in this study and previous known antiviral effects of PARP1 inhibitors reported in the literature, we also discussed several putative mechanisms of the anti-SARS-CoV-2 activities for CVL218 to be involved in the treatment of COVID-19. Overall, our results indicated that the PARP1 inhibitor CVL218 identified by our integrative drug repositioning pipeline may serve as an effective therapeutic agent against COVID-19.

## Results

### Overview of our drug repositioning framework

**Fig. 1:**
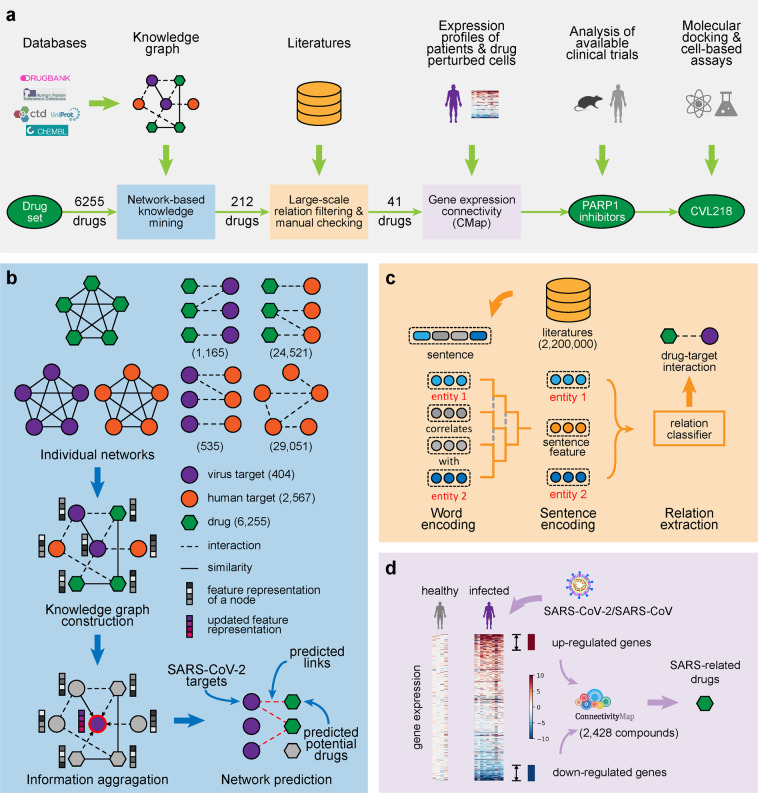
Schematic illustration of our integrative drug repositioning pipeline for discovering the potential drugs to treat the COVID-19 disease. **a** The overview of our drug screening pipeline. The initial drug set for screening contains 6255 drug candidates, mainly including 1786 approved, 1125 investigational, and 3290 experimental drugs. The number of drug candidates after each filtering step is also shown. Knowledge graph: a network containing entities (e.g., drugs and targets) and their relations. **b** The network-based knowledge mining module. Seven individual networks containing three types of nodes (i.e., drugs, human targets and virus targets) and the corresponding edges describing their interactions, associations or similarities are first constructed based on the known chemical structures, protein sequences and relations derived from publically available databases. Then a deep learning based method, which learns and updates the feature representation of each node through information aggregation, is used to predict the potential drug candidates against a specific coronavirus. **c** The automated relation extraction module. The structure of each sentence from the literature texts is first learned from the encoded word features using the Gumbel tree gated recurrent unit technique.^4,96^ Then the learned sequence structures as well as the corresponding encoded word features are fed into a relation classifier to automatically extract the relations between two entities from large-scale documents in the literature. **d** The connectivity map (CMap) analysis module. The transcriptome profiles of the peripheral blood mononuclear cell (PBMC) or the bronchoalveolar lavage fluid (BALF) samples from the SARS-CoV-2 or SARS-CoV infected patients and healthy persons are compared to derive the query gene expression signatures, which are then correlated to the drug-perturbed cellular expression profiles in the connectivity map^5^ to filter out the anti-SARS-CoV-2 drug candidates. For transcriptome data of SARS-CoV-2 infected cells, PBMC samples were provided by three patients and three healthy volunteers, and BALF samples were collected from three patients and two healthy volunteers. For transcriptome data of SARS-CoV infected cells, PBMC samples from ten patients and four healthy volunteers were included in our analysis

The overview of our integrative drug repositioning framework is shown in Fig. 1a. We first constructed a virus-related knowledge graph consisting of drug–target interactions, protein–protein interactions and similarity networks from publically available databases (Methods). Knowledge graph is a network containing entities (e.g., drugs and targets) and their relations. Three different types of nodes (i.e., drugs, human targets, and virus targets) within the knowledge graph were connected through edges describing their interactions, associations or similarities to establish bridges of information aggregation and knowledge mining. We then applied a network-based knowledge mining algorithm, called CoV-DTI, to predict an initial list of drug candidates that can be potentially used to treat SARS-CoV-2 infection (Fig. 1b and Methods). Next, we further narrowed down the list of drug candidates with the previously reported evidences of antiviral activities based on the text-mining results from the large-scale literature texts, which were derived through a deep learning based relation extraction method named BERE^4^ (Fig. 1c and Methods), followed by a minimum of manual checking. After that, we used the connectivity map analysis approach^5^ with the gene expression profiles of SARS-CoV-2 and SARS-CoV infected patients^6,7^ to further refine the list of drug candidates against SARS-CoV-2 (Fig. 1d and Methods).

### Validation of our network-based knowledge mining results

To demonstrate that our computational pipeline for drug repositioning can yield reasonably accurate prediction results, we also validated our network-based knowledge mining algorithm (Fig. 1b) using cross-validation and a retrospective study on the past data of the two coronaviruses that are closely related to SARS-CoV-2 and had been relatively well studied in the literature, i.e., SARS-CoV and MERS-CoV. We first performed ten-fold cross-validation on all virus-related drug–target interactions (DTIs) from our collected data to evaluate the prediction performance of our network-based knowledge mining algorithm CoV-DTI. To mimic a realistic scenario in which the goal is to predict the drug candidates for novel virus targets, we randomly split the virus targets into ten folds and masked the labels of one fold for testing. All the negative samples with unknown DTI labels were used during training when computing the Bayesian personalized ranking loss (Methods). The receiver operation characteristics (ROC) curves and the curves of recall scores with respect to a list of top *k* drug candidates predicted by our model CoV-DTI and DTINet,^8^ our previously developed network-based DTI prediction algorithm which served as a state-of-the-art baseline method, were obtained by averaging the predicted scores from five repeats of ten-fold cross-validation (Supplementary Fig. S1). Our cross-validation results showed that CoV-DTI achieved an area under the ROC (AUROC) score of 0.8273, which was 8% higher than that of DTINet. In addition, over 50% of the drugs known to act on a virus target can be accurately predicted by CoV-DTI from the top 200 drug candidates, with a better performance compared to DTINet. All these cross-validation results demonstrated that our computational method CoV-DTI can make reasonably accurate predictions on the reconstructed virus-related knowledge graph.

In our retrospective study using the past data of SARS-CoV and MERS-CoV, with the aid of our developed text-mining tool BERE, we found that many of the drugs that had been reported previously in the literature to have antiviral activities against the corresponding coronavirus, were also among the top list of our predicted results (Table 1). For example, chloroquine, an FDA-approved drug for treating malaria,^9^ which was previously reported to exhibit micromolar anti-SARS-CoV activity in vitro,^10^ was also repurposed to target the same virus by CoV-DTI. Gemcitabine, which was originally approved for treating certain types of cancers,^11^ was also predicted by CoV-DTI to target SARS-CoV and can be further validated by previous in vitro studies.^12^ Cyclosporine, a calcineurin inhibitor approved as an immunomodulatory drug,^13^ which was previously observed to block the replication of SARS-CoV,^14^ can also be successfully predicted by our approach. Among the top list of predicted drugs against MERS-CoV, miltefosine, which was approved for treating leishmaniasis,^15^ had been previously identified to have anti-MERS-CoV activities.^16^ Chlorpromazine and imatinib, which were used for treating schizophrenia^17^ and leukemia,^18^ respectively, were also selected by CoV-DTI as anti-MERS-CoV drugs and can be validated by previous in vitro evidences.^12^ Thus, the above retrospective study illustrated that our computational framework is able to predict effective drug candidates against a specific coronavirus.

**Table 1:** Selected examples of our predicted drug candidates against SARS-CoV or MERS-CoV that can be validated by the literature evidences in a retrospective study

Drug name	Virus	Original targets^a^	Original indications^b^	References^c^
Chloroquine	SARS-CoV	Fe(II)-protoporphyrin IX (Plasmodium falciparum)	Malaria	^10,102^
Gemcitabine	SARS-CoV	Ribonucleoside-diphosphate reductase large subunit (Human)	Cancer	^12,102^
Cyclosporine	SARS-CoV	Calcineurin subunit B type 2 (Human)	Prophylaxis of organ rejection, severe active rheumatoid arthritis (RA)	^14^
Indomethacin	SARS-CoV	Prostaglandin G/H synthase 1 and 2 (Human)	Symptomatic management of rheumatoid arthritis	^103^
Curcumin	SARS-CoV	Peroxisome proliferator-activated receptor gamma (Human)	Various proinflammatory diseases^104^	^105^
PJ-34	SARS-CoV	Poly-ADP-ribose polymerase 1 (Human)	Experimental allergic encephalomyelitis^106^	^107^
Hesperetin	SARS-CoV	Sterol O-acyltransferase 1 (Human)	Lowering cholesterol^108^	^109^
Miltefosine	MERS-CoV	P-glycoprotein 1 (Human)	Mucosal, cutaneous, visceral leishmaniasis	^16^
Chlorpromazine	MERS-CoV	Dopamine D2 and D1 receptors, 5-hydroxytryptamine receptor 1A and 2A, Alpha-1A and -1B adrenergic receptors, Histamine H1 receptor (Human)	Schizophrenia and other psychotic disorders	^12,102^
Imatinib	MERS-CoV	BCR-ABL fusion kinase (Human)	Leukemia	^102,110^

The drug candidates were first predicted using our network-based knowledge mining algorithm CoV-DTI with a cutoff threshold of *p*-value <0.05. Then the identified drug candidates were validated using an automated relation extraction method from the large-scale literature texts, followed by a minimum of manual checking

^a^The parenthesis indicates the organism of the target(s)

^b^Drug indications stand for the official indications approved by the FDA, obtained from DrugBank,^19^ unless other references are stated

^c^References stand for the supporting literatures

### PARP1 inhibitors as potential drug candidates for COVID-19

Through careful examination of the screening results derived mainly by CoV-DTI and BERE and the connectivity map analysis results derived using the transcriptomic profiles of SARS-CoV-2/SARS-CoV-infected patients, we found that poly-ADP-ribose polymerase 1 (PARP1) inhibitors (Supplementary Fig. S2), such as PJ-34 and olaparib, were highlighted as potential therapeutic agents that may have the antiviral activities against SARS-CoV-2 (Table 2, Supplementary Table S1 and Supplementary Table S2). We then focused on this drug class and selected those drug candidates with both potential efficacy and acceptable pharmacokinetic and toxicological profiles for further investigation. We first noticed that PJ-34 was the most preferred drug derived from our computational framework, as both our network-based knowledge mining algorithm CoV-DTI and connectivity map analysis pointed to this drug (Table 2 and Supplementary Table S1). After checking the clinical status of PJ-34, we found that it only reached the preclinical trial stage (DrugBank ID: DB08348,^19^) which may raise the concern about its safety issue and thus limit its timely application for the current clinical usage against COVID-19. Therefore, we compared the molecular characteristics between PJ-34 and other PARP1 inhibitors that had been already approved by the Food and Drug Administration (FDA) or at least entered the clinical trials (Methods), and found that rucaparib and CVL218 (mefuparib) share the highest similarities with PJ-34. Considering the poor accessibility of rucaparib in the lung tissue (https://www.ema.europa.eu/en/medicines/human/EPAR/rubraca), it may be hard to apply this small molecule in treating the pneumonia caused by SARS-CoV-2. Thus, we mainly selected CVL218 to conduct further experiments, and this drug was later validated to exhibit a much better tissue distribution with high concentration in lung (see Supplementary Materials for more details). We also included olaparib, the first FDA-approved PARP1 inhibitor, in our further analyses, as this drug also achieved a high rank in our connectivity map analysis using the transcriptomic data of COVID-19 patients (Table 2).

**Table 2:** The top list of drug candidates identified by our connectivity map analysis using the gene expression profiles of the peripheral blood mononuclear cell (PBMC) samples of three SARS-CoV-2 infected patients, the bronchoalveolar lavage fluid (BALF) samples of two SARS-CoV-2 infected patients^6^ and the PBMC samples of ten SARS-CoV infected patients^7^

Connectivity Map Score	Compound BRD ID	Name	Description
ARS-CoV-2 (PBMC)			
−99.02	BRD-K07762753	Aminopurvalanol-a	Tyrosine kinase inhibitor
−98.59	BRD-K50836978	Purvalanol-a	CDK inhibitor
−98.39	BRD-K02113016	**Olaparib**	**PARP inhibitor**
−96.77	BRD-K69932463	AZD-8055	MTOR inhibitor
−96.35	BRD-K67566344	KU-0063794	MTOR inhibitor
−95.86	BRD-K98490050	Amsacrine	Topoisomerase inhibitor
−95.74	BRD-K04546108	JAK3-inhibitor-VI	JAK inhibitor
−95.56	BRD-K56334280	Amonafide	Topoisomerase inhibitor
−95.53	BRD-A82371568	Clofarabine	Ribonucleoside reductase inhibitor
−95.42	BRD-K12184916	Dactolisib	MTOR inhibitor

SARS-CoV-2 (BALF)			
−98.38	BRD-K39569857	Avrainvillamide-analog-3	nucleophosmin inhibitor
−97.78	BRD-K00615600	**AG-14361**	**PARP inhibitor**
−97.67	BRD-K50387473	XMD-892	MAP kinase inhibitor
−96.65	BRD-U51951544	ZG-10	JNK inhibitor
−94.99	BRD-K00317371	RITA	MDM inhibitor
−94.48	BRD-K68191783	ALW-II-38-3	Ephrin inhibitor
−94.29	BRD-A39646320	HC-toxin	HDAC inhibitor
−93.98	BRD-K54256913	MK-1775	WEE1 kinase inhibitor
−92.84	BRD-K87932577	CDK1-5-inhibitor	CDK inhibitor
−90.73	BRD-A29901043	KIN001-127	ITK inhibitor

SARS-CoV (PBMC)			
−98.94	BRD-K87142802	**Veliparib**	**PARP inhibitor**
−95.37	BRD-A35338386	NECA	Adenosine receptor agonist
−95.26	BRD-K11853856	**PJ-34**	**PARP inhibitor**
−92.96	BRD-A53952395	Prilocaine	Local anesthetic
−91.80	BRD-K32977963	Eugenol	Androgen receptor antagonist
−91.56	BRD-A09495397	Bicuculline	GABA receptor antagonist
−91.32	BRD-K82164249	Andarine	Androgen receptor modulator

The default connectivity map score^5^ of −90.0 was used as the cutoff threshold to determine the top list, i.e., only those drug candidates with the connectivity scores of the query ranked to the top 10% of the reference perturbations were selected. PARP1 inhibitors (i.e., olaparib, veliparib, and PJ-34) were chosen into the top list (shown in bold). For the connectivity map analysis on the gene expression data of SARS-CoV-2 infected patients, only top 10 drug candidates are listed below, and the remaining drug candidates are shown in Supplementary Table S2

### CVL218 exhibits in vitro inhibitory activity against SARS-CoV-2 replication

**Fig. 2:**
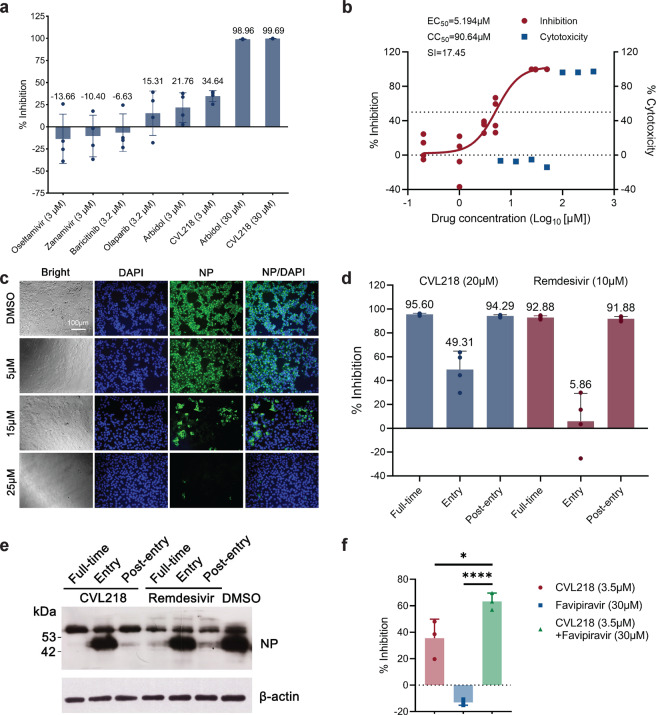
The in vitro anti-SARS-CoV-2 activities of the tested drugs in Vero E6 cells. **a** The preliminary in vitro antiviral activities of oseltamivir at 3 μM, zanamivir at 3 μM, baricitinib at 3.2 μM, olaparib at 3.2 μM, arbidol at 3 μM and 30 μM, and CVL218 at 3 μM and 30 μM, respectively, were detected in Vero E6 cells infected with SARS-CoV-2 at an MOI of 0.05. The viral yield in the cell supernatant was then quantified by qRT-PCR. Results are shown as mean ± SD over four replicates. **b** The concentration-dependent inhibition curve of CVL218 against SARS-CoV-2 replication and its cytotoxicity results. Viral infection and drug treatment at different concentrations were performed as mentioned above. Cytotoxicity of CVL218 to Vero E6 cells was measured by the CCK8 assays. **c** Visualization of virus nucleoprotein (NP) expression of the infected cells upon treatment of CVL218 at 48 h post the SARS-CoV-2 infection using fluorescence microscopy. **d** Time-of-addition results on the inhibition of CVL218 and remdesivir against SARS-CoV-2 in vitro. The viral inhibitory activities of CVL218 and remdesivir were measured at “full-time”, “entry”, and “post-entry” stages, respectively. Results are shown as mean ± SD over four replicates. **e** Virus NP expression in the infected cells upon the treatment of CVL218 and remdesivir was analyzed by western blot. **f** In vitro inhibitory activities against SARS-CoV-2 replication of favipiravir (30 μM), CVL218 (3.5 μM) and a combination of both drugs (30 μM favipiravir + 3.5 μM CVL218). The concentrations were selected according to the EC_25_ values of individual drugs against SARS-CoV-2 in vitro. Viral infection and drug treatment were performed as mentioned above. Results are shown as mean ± SD over three replicates, and the significances were measured by *p*-values from *t*-tests. * and **** stand for *p*-value < 0.05 and *p*-value < 0.0001, respectively

We first conducted a pilot experimental test in vitro (Methods) on the anti-SARS-CoV-2 activities of CVL218, olaparib and several other related drugs (Fig. 2a). We found that both PARP1 inhibitors olaparib and CVL218 exhibited inhibitory effects against SARS-CoV-2 replication. Nevertheless, CVL218 showed a much higher inhibition rate than olaparib. More specifically, olaparib inhibited SARS-CoV-2 replication by 15.31% at a concentration of 3.2 μM, while CVL218 reached 34.64% reduction at a concentration of 3 μM.

Notably, the antiviral efficacy of CVL218 even surpassed arbidol, which has been used in clinical studies for the treatment of COVID-19.^20^ In particular, arbidol inhibited SARS-CoV-2 replication by 21.76% at 3 μM, much lower than that of CVL218 at the same concentration (Fig. 2a). In contrast, oseltamivir, zanamivir (drugs used for preventing influenza virus infection), and baricitinib (JAK1/2 inhibitor, which was recommended in ref. ^21^ to treat COVID-19) showed no inhibitory activities against SARS-CoV-2 at the concentration of 3 μM or 3.2 μM.

Based on the above preliminary results in the pilot experimental test, we then chose CVL218 for subsequent experimental studies. Our further in vitro assays (Methods) showed that CVL218 exhibited effective inhibitory activity against SARS-CoV-2 replication in a dose-dependent manner, with an EC_50_ of 5.194 μM (Fig. 2b). We also assessed the cytotoxicity of CVL218 by the CCK8 assay (Methods), and found that CVL218 had a CC_50_ of 90.64 μM in Vero E6 cells. Furthermore, immunofluorescence microscopy (Methods) revealed that, at 14 h post SARS-CoV-2 infection, virus nucleoprotein (NP) expression in the CVL218-treated cells demonstrated a dose-response relationship with the treated drug concentrations, and was significantly lower upon CVL218 treatment compared to that in the DSMO treated cells (Fig. 2c). Lower expression level of N gene compared to DMSO was also observed through RT-PCR experiments (Supplementary Fig. S3). In addition, no obvious cytopathic effect was observed in the infected cells treated with CVL218 at 25 μM (Fig. 2c).

To systematically assess the inhibitory activities of CVL218 against SARS-CoV-2, we also performed a time-of-addition assay (Methods) to determine at which stage CVL218 inhibits viral infection. Remdesivir, which has entered the clinical trials for the treatment of COVID-19 (https://clinicaltrials.gov/ct2/show/NCT04257656), was also tested in this assay for comparison. In particular, compared to the DMSO control group, both CVL218 and remdesivir showed potent antiviral activities during the full-time procedure of SARS-CoV-2 infection in Vero E6 cells (Fig. 2d). In addition, this time-of-addition assay indicated that CVL218 can partially work against the viral entry and significantly inhibit the replication of virus post-entry, while the remdesivir can only function at the post-entry stage (Fig. 2d, e). All together, results of the above in vitro assays indicated that CVL218 can be considered a potential therapeutic agent for treating COVID-19.

We further examined the drug combination effect of CVL218 with another anti-SARS-CoV-2 drug candidate favipiravir, which was originally designed to target the RNA-dependent RNA polymerase (RdRp) of influenza A virus,^22^ and had been recently reported to exhibit in vitro anti-SARS-CoV-2 activity with an EC_50_ of 61.88 μM^23^ and a higher 7-day recovery rate than arbidol in clinical trials for treating COVID-19.^24^ In particular, the inhibitory activities against SARS-CoV-2 replication of favipiravir at 30 μM, CVL218 at 3.5 μM and a combination of both were measured in Vero E6 cells (Methods). We found that, while favipiravir exhibited no inhibitory activity and CVL218 showed an inhibition rate of 36% at the experimental concentrations, the combination of both CVL218 and favipiravir achieved a significantly higher inhibition rate (64%, Fig. 2f). This result indicated that CVL218 can also be potentially combined with other drug candidates to enhance the therapeutic efficacy against SARS-CoV-2.

### CVL218 interacts with the nucleocapsid protein of SARS-CoV-2

**Fig. 3:**
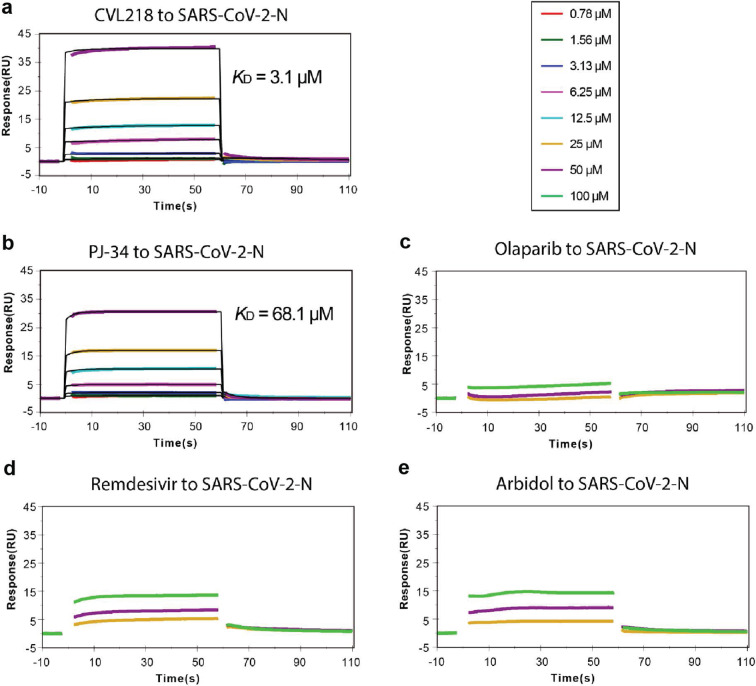
CVL218 binds to the nucleocapsid protein of SARS-CoV-2 (SARS-CoV-2-N). **a**–**e** Kinetic analyses of the binding between SARS-CoV-2-N and the tested drugs, including CVL218 (**a**), PJ-34 (**b**), olaparib (**c**), remdesivir (**d**), and arbidol (**e**), measured by a SPR-based Biacore instrument. The derived dissociation constants between SARS-CoV-2-N and the tested drugs (CVL218 and PJ-34) are also shown

To further investigate the potential target of SARS-CoV-2 that CVL218 acts on, we next performed an in vitro surface plasmon resonance (SPR) assay to measure its binding affinity with the nucleocapsid (N) protein of SARS-CoV-2 (SARS-CoV-2-N) (Fig. 3, Methods). In this SPR assay, we also tested another two PARP1 inhibitors, including PJ-34 and olaparib, and two related anti-SARS-CoV-2 drugs, including remdesivir and arbidol, for their possible binding to SARS-CoV-2-N. As expected, no binding was observed for remdesivir and olaparib (Fig. 3d, e). This result was consistent with the known facts that remdesivir mainly performs its antiviral activity by targeting the RNA-dependent RNA polymerase (RdRp) of coronavirus^25^ and arbidol was originally designed to inhibit the virus-host cell fusion against influenza virus.^26,27^ Among all the three tested PARP1 inhibitors, including CVL218, PJ-34 and olaparib, CVL218 exhibited the highest binding strength towards SARS-CoV-2-N with a dissociation constant (*K*_*D*_) of 3.1 μM (Fig. 3a). On the other hand, PJ-34 displayed a much lower binding affinity to SARS-CoV-2-N (*K*_*D*_ = 68.1 μM), which was about 22-fold lower than that of CVL218 (Fig. 3b). Surprisingly, we observed no binding signal with SARS-CoV-2-N for the PARP1 inhibitor olaparib (Fig. 3c). All these binding results derived from our SPR assay demonstrated that CVL218 interacts with SARS-CoV-2-N with a higher binding affinity than the other two tested PARP1 inhibitors, implying a potential antiviral mechanism mediated by the interplay between CVL218 and the nucleocapsid protein of SARS-CoV-2.

### CVL218 inhibits the LPS-induced production of cytokines in PBMCs

The inflammatory cytokines are heavily involved in the responses to viral infections,^28,29^ and their excessive production can be directly associated with the pathogenesis of the corresponding diseases.^30–32^ On the one hand, the extra synthesis of cytokines may promote to viral infection. For example, a previous study showed that IL-6 blockage can reduce viral loads and enhance virus-specific CD8+ T-cell immunity in the Friend retrovirus (FV) mouse model.^33^ In addition, it was previously reported that IL-10 can facilitate West Nile virus (WNV) infection in mice, and its knockout can diminish the WNV infection.^34^ On the other hand, the overproduction of cytokines may cause inflammatory injuries to human tissues. For example, the respiratory syncytial virus (RSV) infection can promote the upregulation of TNF-*α* and IFN-*γ*, and thus lead to airway inflammation and lung immune injuries.^35,36^ Moreover, the excessive synthesis of cytokines was found to be highly related with the deleterious clinical manifestations under the infection conditions of many other viruses, such as tick-borne virus (TBV),^37^ influenza A virus (IAV),^38^ and human immunodeficiency virus (HIV).^39^ These findings indicated that inhibiting the overproduction of cytokines may offer a beneficial strategy to protect against viral infections.^40^

Recently, a number of studies on the clinical characteristics of severe COVID-19 patients had shown that several proinflammatory cytokines, including IL-6, IL-10, IFN-*γ*, TNF-*α,* and others, are significantly elevated especially in those ICU patients infected by SARS-CoV-2, causing excessive activated immune response.^41–45^ The pathological relevance of these cytokines in SARS-CoV-2 infection indicated that their blockade may alleviate the inflammatory response and thus provide a feasible therapy for the treatment of COVID-19.

**Fig. 4:**
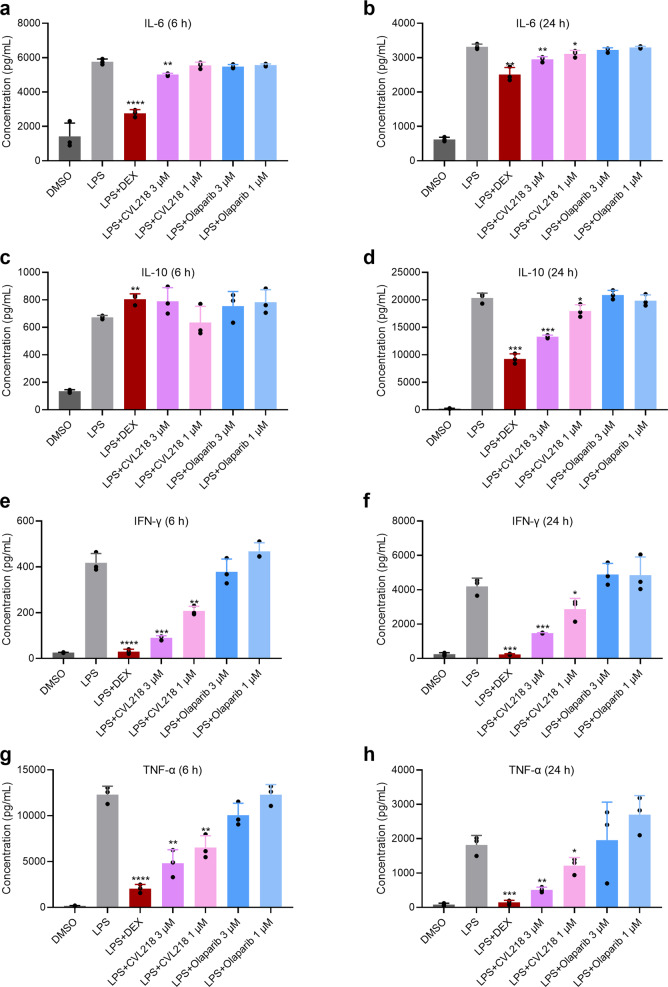
CVL218 attenuates the LPS-induced cytokine production in a time- and dose-dependent manner. Concentrations of four cytokines (IL-6, IL-10, IFN-*γ*, and TNF-*α*) in the LPS-induced peripheral blood mononuclear cells (PMBCs) were measured by ELISA after 6 h and 24 h of drug treatment. Dexamethasone (DEX) in 1 μg/mL was used as a positive control. **a**–**h** Concentrations of IL-6 after 6 h (**a**) and 12 h (**b**), IL-10 after 6 h (**c**) and 12 h (**d**), IFN-*γ* after 6 h (**e**) and 12 h (**f**), and TNF-*α* after 6 h (**g**) and 12 h (**h**) of drug treatment. Results are shown as mean ± SD over three replicates. The significances were measured by *p*-values from two-*t*ailed *t*-tests between LPS+ drug and LPS only groups. *, **, ***, and **** stand for *p*-value < 0.05, 0.01, 0.001, 0.0001, respectively

To test whether our identified drug CVL218 is able to regulate the expression of cytokines in vitro, we first stimulated the cytokine production of the peripheral blood mononuclear cells (PMBCs) by lipopolysaccharide (LPS) and then measured the concentrations of four cytokines (i.e., IL-6, IL-10, IFN-*γ*, and TNF-*α*) that are highly relevant to the pathogenesis of COVID-19 after 6 h and 24 h of drug treatment. As shown in Fig. 4, the production of these four cytokines were all significantly elevated in the LPS-induced PBMCs. In the presence of 3 μM CVL218, the LPS-induced production of IL-6 was significantly repressed after both 6 h and 24 h incubation (*p*-values < 0.01 for both timepoints, Fig. 4a, b). Although the LPS-induced expression of IL-10 was not downregulated by 3 μM CVL218 treatment after 6 h, it was dramatically reduced after 24 h (*p*-value < 0.001), indicating that IL-10 may responde to CVL218 treatment in a time-dependent manner (Fig. 4c, d). Both 3 μM and 1 μM CVL218 after 6 h and 24 h of drug treatment led to a significant decrease in the concentrations of IFN-*γ* and TNF-*α* compared to their original expression induced by LPS (*p*-values < 0.01 for the 3 μM groups and *p*-values < 0.05 for the 1 μM groups, Fig. 4e–h). In addition, the inhibitory effects of 1 μM CVL218 were generally weaker than those of 3 μM CVL218, indicating the dose-dependent inhibition of CVL218 on the LPS-induced expression of cytokines. In comparison, the olaparib treatment at both 3 μM and 1 μM exhibited no significant inhibition to the LPS-induced stimulation of all four cytokines. All these results demonstrated that CVL218 can significantly suppress the LPS-induced elevation of cytokines in a dose- and time-dependent manner, and thus provided an in vitro evidence to support CVL218 as a potential therapeutic agent for targeting the proinflammatory response caused by SARS-CoV-2 infection.

## Discussion

In this study we reported a top-down integrative drug repositioning approach by combining both machine learning and statistical analysis techniques, followed by manual selection, to identify potential drug candidates against SARS-CoV-2. We showed that the PARP1 inhibitor CVL218 discovered by our integrative framework exhibits effective anti-SARS-CoV-2 activity in vitro and thus can be used as a potential drug candidate for treating COVID-19. We also validated that CVL218 can interact with the N protein of SARS-CoV-2, indicating that CVL218 may inhibit viral replication mainly through acting on the N protein and thus impeding its normal functions. To our best knowledge, this is the first antiviral drug candidate proposed to target the N protein of SARS-CoV-2, which thus may provide a novel mechanistic solution to the treatments of COVID-19. Benefited from the distinct antiviral mechanism of CVL218 compared to other drug candidates targeting at COVID-19, we proposed that the efficacy of CVL218 against SARS-CoV-2 infection can be enhanced by combinatorial usage with other drug candidates, and experimentally validated the drug combination effect between CVL218 and favipiravir.

While our paper was under review, a new dataset of human protein-virus protein interactions had been published.^46^ We further tested our model on an updated knowledge graph constructed by incorporating this new dataset. Our model achieved a slightly better performance on the updated knowledge graph (Supplementary Table S3), which indicated that our prediction results can be further improved by incorporating more information.

**Fig. 5:**
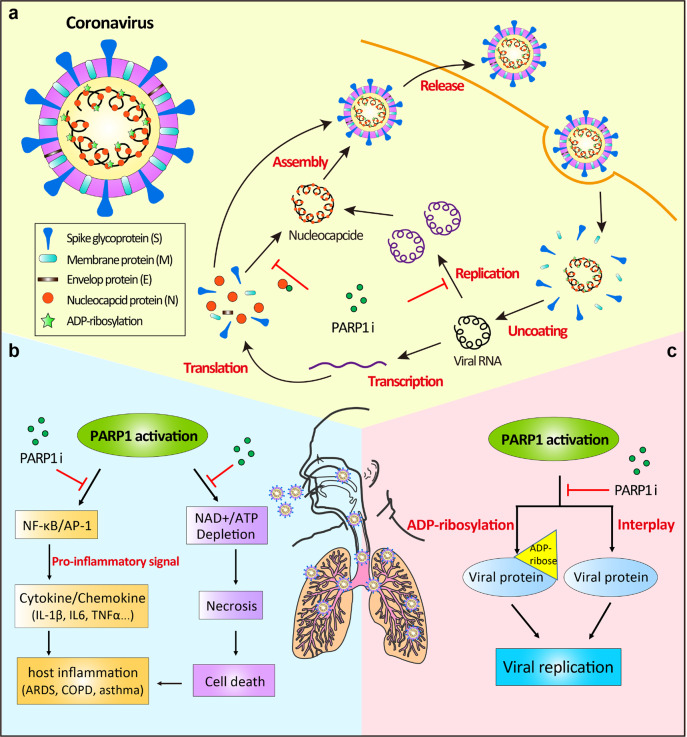
The putative mechanisms for CVL218 as a PARP1 inhibitor to combat the COVID-19 disease, derived based on the data present in this study and the known antiviral activities of PARP1 inhibitors previously reported in the literature. **a** Schematic diagram showing the possible antiviral mechanisms of PARP1 inhibitors in the life cycle of coronavirus in human cells. PARP1 inhibitors have been previously reported in the literature to suppress viral replication and imped the binding of nucleocapsid protein to viral RNAs, thus preventing the virus infection.^47–50^ **b** Potential protective effects of PARP1 inhibitors in the treatment of COVID-19. The anti-inflammation effects of PARP1 inhibitors may be achieved through two possible molecular pathways. The first one is to modulate the expression of pro-inflammation factors such as NF-*κ*B, AP-1, IL-6 and downstream cytokines and chemokines.^51–54^ The second possible pathway is to prevent the overactivation of PARP1 and thus avoid the depletion of NAD^+^ and ATP, and the consequent cellular energy failure and cell death caused by necrosis.^51–54^ **c** The potential antiviral effects of PARP1 inhibitors through suppressing the ADP-ribosylation of viral proteins and intervening the host-pathogen interactions, thus resulting in the inhibition of viral replication^48,49,56,57^

Based on the data present in this study and the previously known evidences about the antiviral effects of PARP1 inhibitors reported in the literature, we propose several potential mechanisms to support the involvement of the CVL218 in the treatment of COVID-19 (Fig. 5). First, it has been known that, during the life cycle of the coronavirus, PARP1 inhibitors may inhibit the viral growth through suppressing viral replication and impeding the binding of the nucleocapsid protein to viral RNAs,^47–50^ which can also be supported by our SPR assay results. Second, PARP1 inhibitors have been previously reported to play a critical role in regulating the inflammatory response by modulating the expression of proinflammatory factors such as NF-*κ*B, AP-1, IL-6, TNF-*α* and downstream cytokines and chemokines.^51–54^ Also, it has been shown that the overactivation of PARP1 promotes energy (NAD^+^/ATP) consumption and drives cell death, exacerbating the inflammation response.^51–53,55^ PARP1 inhibitors thus may repress the NF-*κ*B-mediated proinflammatory signals, and reduce energy failure and subsequent cell death induced by necrosis, hence providing a clinical potential for attenuating the cytokine storm and inflammatory response caused by SARS-CoV-2 infection. Third, ADP-ribosylation is a conserved post-translational modification on the nucleocapsid proteins across different coronavirus lineages, implying that it may have an important regulatory role for the structure packing of viral genome. Several previous studies have demonstrated that PARP1 is critical for viral replication.^49,56,57^ For example, PARP1 has been reported to interact with hemagglutinin (HA) of influenza A virus (IAV) and promote its replication by triggering the degradation of host type I IFN receptor.^58^ In addition, the ADP-ribosylation of adenoviral core proteins has been shown to display an antiviral defense mechanism.^48^ Therefore, intervening the ADP-ribosylation mediated interplay between PARP1 and viral proteins may be another important pathway for PARP1 inhibitors to prevent SARS-CoV-2 infection. Of course, to thoroughly understand the anti-SARS-CoV-2 roles of CVL218 and other PARP1 inhibitors, more experimental studies and direct (clinical) evidences will be needed in the future.

According to our qRT-PCR experiment (Supplementary Fig. S3), the downregulation of NP expression was due to the decrease in RNA level. This can be explained by multiple potential mechanisms, including the direct interaction between CVL218 and viral N protein (Fig. 5a) or the inhibition of PARP activity by the compound (Fig. 5c). Although the second hypothesis cannot be excluded without further experimental validation on the effects of PARP1 deficiency on NP expression, we propose that the first hypothesis (i.e., direct CVL218-NP interaction inhibits viral RNA replication) mainly accounts for the downregulation of NP expression by CVL218 treatment, mainly based on our experimental results. In particular, our antiviral assays demonstrated that only CVL218 but not olaparib inhibited viral replication (Fig. 2a), suggesting that the antiviral effect was likely due to a mechanism which is not shared by both compounds. While both CVL218 and olaparib are known to inhibit PARP activity, only CVL218 was shown to bind NP by our SPR experiments (Fig. 3). According to the above reasoning, it is more likely that CVL218 regulated the NP expression through direct interaction with NP and thus suppressing viral RNA assembly and replication.

Considering the proinflammatory role of PARP1, the therapeutic effects of PARP1 inhibitors in inflammatory-mediated diseases have been extensively studied over past two decades.^59–61^ A first generation PARP1 inhibitor, 3-aminobenzamide, was observed to protect against lung inflammation by reducing NF-*κ*B activity and IL-8 expression.^62^ PJ-34, a second generation PARP1 inhibitor, has been suggested in previous studies to have neuroprotective effects in a stroke model and protect mice from necroptosis-associated liver injuries by repressing the IL-33 expression.^63,64^ Numerous preclinical studies demonstrated that PARP1 inhibitors play an essential role in a range of inflammatory injuries and related diseases, especially the lung inflammatory disorders including ARDS (Acute Respiratory Distress Syndrome), COPD (Chronic Obstructive Pulmonary Disease) and asthma.^54,60,65,66^ All these studies suggest that PARP1 inhibitors are of high relevance to the treatment of the novel pneumonia caused by SARS-CoV-2 infection, possibly via their roles in modulating inflammatory response.

Notably, current pathological studies have shown that the severe patients infected by SARS-CoV-2 generally have higher plasma levels of IL-2, IL-6, IL-10, TNF-*α*, IFN-*γ,*^41,43–45^ implying a high risk of the inflammatory-associated cytokine storm after viral infection. In addition, reduction and functional exhaustion of T cells have also been observed in COVID-19 patients.^43^ Therefore, blocking the overactive inflammatory response may serve as an effective strategy for the treatment of COVID-19, particularly for those ICU patients infected by SARS-CoV-2. According to a recent study, both innate and adaptive immune responses are activated in COVID-19 patients and the overproduction of cytokines like IL-6 and TNF-*α* is likely to be involved in cytokine storm syndrome (CSS).^67^ Figuring out the specific immune cell subsets which these cytokines belong to could benefit our understanding about the pathology of severe COVID-19 patients and design of novel therapies targeting this disease. Recently, tocilizumab, a monoclonal antibody drug targeting IL-6, has been shown to have therapeutic potential for the treatment of COVID-19,^68^ which also highlights the vital role of anti-inflammatory response in current therapeutics against SARS-CoV-2. Our in vitro study has shown that CVL218 can effectively inhibit the production of several inflammatory cytokines induced by LPS in PBMCs, including IL-6, IL-10, IFN-*γ*, and TNF-*α*, which are highly related to the pathogenic characteristics of COVID-19 (Fig. 4). This finding indicates that CVL218 may also possess a good anti-inflammatory profile that is specifically applicable to those severe patients infected by SARS-CoV-2.

As shown in Fig. 4, CVL218 exhibited significant activity of anti-inflammatory effects while another PARP inhibitor olaparib did not show such an effect. One possible reason is that CVL218 exhibited better selectivity to the members of PARP family compared with olaparib,^69–71^ and thus CVL218 and olaparib may modulate different regulatory pathways of the inflammatory response. In addition, it still remains controversial whether the enzymatic activity of PARP1 is required for activating NF-*κ*B.^72–74^ Collectively, we hypothesize that the PARP1 inhibitors CVL218 and olaparib may play different roles in regulating the inflammatory pathways.

PARP1 inhibitors are originally used for targeting the homologous recombination repair defects in cancers, and mainly categorized as oncology drugs. Thus, it would generally need more safety data to justify any repurposing of PARP1 inhibitors for non-oncology indications. Fortunately, there are numerous existing preclinical and clinical studies on repurposing PARP1 inhibitors into non-oncological diseases, including the aforementioned acute diseases (e.g., acute respiratory distress syndrome (ARDS), stroke)^75^ and chronic diseases (e.g., rheumatoid arthritis and vascular diseases).^75,76^ All these evidences indicate the possibility of repurposing PARP1 inhibitors as a safe therapeutic agent to treat the current acute lung disease caused by SARS-CoV-2 infection. In addition, the pharmacokinetic and toxicokinetic data in rats and monkeys shown in our study indicate that CVL218 possesses an acceptable safety profile to be repositioned for the anti-SARS-CoV-2 purpose. Moreover, CVL218 has been approved to enter Phase I clinical trial in 2017 for cancer treatment (Registration Number: CTR20190906). The preliminary data from the Phase I clinical trial have shown that CVL218 is well tolerated in ascending dose studies at doses as high as 1000 mg QD and 500 mg BID, and no Grade II and above adverse events have been observed, which indicates that CVL218 is also quite safe and well tolerated in human.

Our pharmacokinetic examination in rats has shown that CVL218 has the highest tissue distribution in lung, with a 188-fold higher concentration compared to that in plasma. Such a tissue specific enrichment in lung may bring an extra advantage for CVL218 to be used for the anti-SARS-CoV-2 purpose, as lung is the therapeutically targeted tissue for COVID-19. Moreover, the high-level distribution in lung may also suggest that only a low dosage is needed to ensure the therapeutic efficacy of CVL218 against SARS-CoV-2, which may further reduce the risk of adverse events. Thus, CVL218 may have a great potential to be repurposed as an effective therapeutic agent to combat SARS-CoV-2 and prevent the future epidemic outbreak.

## Materials and methods

### Construction of the virus-related knowledge graph

The virus-related knowledge graph was constructed for predicting the coronavirus-related drugs. In total seven networks were considered in the constructed knowledge graph (Fig. 1b), including a human target–drug interaction network, a virus target–drug interaction network, a human protein–protein interaction network, a virus protein-human protein interaction network, a drug molecular similarity network, a human protein sequence similarity network, and a virus protein sequence similarity network. The human target–drug interaction network was derived from DrugBank (version 5.1.0).^19^ The virus target–drug interaction network was constructed from the integrated data from DrugBank (version 5.1.0),^19^ ChEMBL (release 26),^77^ TTD (last update 11 Nov, 2019),^78^ IUPHAR_BPS (release 13, Nov, 2019),^79^ BindindDB^80^ and GHDDI (https://ghddi-ailab.github.io/Targeting2019-nCoV/CoV_Experiment_Data/), with a cutoff threshold of IC_50_/EC_50_/K_*i*_/K_*d*_<10 *μ*M. The human protein–protein interaction network and the virus protein-human protein interaction network were constructed from the integrated data from BioGRID (release 3.5.181),^81^ HuRI,^82^ Instruct,^83^ MINT (2012 update),^84^ PINA (V2.0),^85^ SignaLink (V2.0)^86^ and innatedb.^87^ The drug molecular similarity network was obtained by calculating the Tanimoto similarities from Morgan fingerprints with a radius of 2 computed using the rdkit tool.^88^ The protein sequence similarity networks of both human and virus were obtained by calculating the Smith-Waterman similarities of the amino acid sequences derived from UniProt^89^ using a sequence alignment software provided in.^90^ Noted that we collected additional protein sequences of SARS-CoV-2 from UniProt^89^ and added them into the corresponding networks for the final prediction. Those drugs without drug–target interactions or outside the DrugBank database were removed from the corresponding networks. We then constructed the virus-related knowledge graph by merging together all the nodes and edges of the above seven networks (Fig. 1b). The constructed knowledge graph *G* = (*V*, *E*) is an undirected graph, in which each node *v* ∈ *V* in the node set *V* belongs to one of the node types (including drugs, human proteins, and virus proteins), and each edge *e* ∈ *E* in the edge set *E* ⊂ *V* × *V* × *R* belongs to one of the relation types from the relation type set *R* (including two drug–target interactions, two protein–protein interactions and three similarities).

### The network-based knowledge mining algorithm

The initial list of drug candidates targeting SARS-CoV-2 was first screened using a network-based knowledge mining algorithm, called CoV-DTI, which was modified from our previous work.^8,91^ The goal of CoV-DTI is to capture the hidden virus-related feature information and accurately predict the potential drug candidates from the constructed knowledge graph, which is realized through learning a network topology-preserving embedding for each node.

More specifically, CoV-DTI uses a graph convolution algorithm^92^ to gather and update feature information for each node in the constructed heterogeneous knowledge graph network from neighborhoods so that the network topology information can be fully exploited. Suppose that we perform *T* iterations of graph convolution. At iteration 1 ≤ *t* ≤ *T*, the message $m_v^t$ passed to node *v* can be expressed as:

1 $$\begin{array}{*{20}{c}} {{\mathbf{m}}_v^t = \mathop {\sum }\limits_{r \in R} \mathop {\sum }\limits_{\begin{array}{*{20}{c}} {u \in N_r\left( v \right),} \\ {e = \left( {u,v,r} \right) \in E} \end{array}} A_{u,v,r}ReLU\left( {{\mathbf{W}}_r^th_u^{t - 1} + {\mathbf{b}}_r^t} \right),} \end{array}$$

where *a*_*v,u,r*_ stands for the weight for edge *e* = (*u, v, r*), $A_{u,v,r} = \frac{{a_{v,u,r}}}{{\mathop {\sum }\nolimits_u a_{v,u,r}}}$, ${\mathbf{W}}_r^t \in {\Bbb R}^{d \times d}$, and ${\mathbf{b}}_r^t \in {\Bbb R}^d$ stand for the learnable parameters, $ReLU(x) = {\mathrm{max}}(0,x)$, and $N_r(v) = \{ u,\,u \in V,\,u \,\ne\, v,\,(u,v,r) \in E\}$ denotes the set of adjacent nodes connected to *v* ∈ *V* through edges of type *r* ∈ *R*.

Then the feature $h_v^t$ of node *v* is updated by

2 $$\begin{array}{*{20}{c}} {h_v^t = \frac{{ReLU\left( {{\mathbf{W}}^t{\mathrm{concat}}\left( {h_v^{t - 1},{\mathbf{m}}_v^t} \right) \,+\, h_v^{t - 1} \,+\, {\mathbf{b}}^t} \right)}}{{\left\| {ReLU({\mathbf{W}}^t{\mathrm{concat}}(h_v^{t - 1},{\mathbf{m}}_v^t) \,+\, h_v^{t - 1} \,+\, {\mathbf{b}}^t)} \right\|_2}},} \end{array}$$

where ${\mathbf{W}}^t \in {\Bbb R}^{d \times d}$ and ${\mathbf{b}}^t \in {\Bbb R}^d$ stand for the learnable parameters, and *concat* (·,·) stands for the concatenation operation.

Finally, the confidence score *s*_*u,v*_ of the relation *r* between node *u* and node *v* is derived from the learned node embeddings and the corresponding projection matrices, that is,

3 $$\begin{array}{*{20}{c}} {s_{u,v} = h_u^{t\top} \cdot G_r \cdot H_r^\top \cdot h_v^t,} \end{array}$$

where $G_r,H_r \in {\Bbb R}^{d \times k}$ stand for the edge-type-specific projection matrices.

CoV-DTI minimizes the Bayesian personalized ranking (BPR) loss^93^ for drug–target interaction reconstruction, by regarding those edges not in the edge set *E* as missing values rather than negative samples, that is,

4 $$\begin{array}{*{20}{c}} {\mathop {\sum }\limits_{r \in R} \mathop {\sum }\limits_{\begin{array}{*{20}{c}} {u,v,w,x \in V,} \\ {\left( {u,v,r} \right) \in E,} \\ {\left( {w,x,r} \right) \notin E} \end{array}} - {\mathrm{log}}\left[ {\sigma \left( {s_{u,v} - s_{w,x}} \right)} \right],} \end{array}$$

where, *s*_*u, v*_ and *s*_*w, x*_ stand for the confidence scores of the relation *r* between *u* and *v* and between *w* and *x*, respectively, and *σ*(·) stands for the sigmoid activation function. Intuitively, in the above loss function, the confidence scores of the node pairs (*u, v*) in the edge set (i.e., (*u*, *v*, *r*) ∈ *E*) are encouraged to be higher than those of unseen pairs (*w*, *x*) (i.e., (*w*, *x, r*) ∉ *E*).

We predicted the confidence scores under the relation of virus target–drug interactions for each virus target–drug pair using Eq. (3). Then the confidence scores were averaged across all the proteins of a certain virus (e.g., SARS-CoV, MERS-CoV, or SARS-CoV-2), and the corresponding *p*-values were obtained by z-test. For each virus, we selected those predictions with a *p*-value < 0.05 as drug candidates.

### Automated relation extraction from large-scale literature texts

We used a deep learning based relation extraction method named BERE^4^ to extract the coronavirus-related drugs from large-scale literature texts. More specifically, the sentences mentioning the two entities of interest, i.e., name (or alias) of a coronavirus or coronavirus target, or name (or alias) of a drug, are first collected using a dictionary-based name entity recognition method (string matching). For each pair of entities (*e*_1_, *e*_2_), there are usually more than one sentence describing the underlying relations. Therefore, we use a bag of sentences $S_{e_1,e_2}$, denoting the set of all the sentences mentioning both *e*_1_ and *e*_2_, to predict the relation between these two entities.

We first encode each sentence $s \in S_{e_1,e_2}$ in a semantic and syntactic manner using a hybrid deep neural network ($h:s \to {\Bbb R}^d$), including a self-attention module,^94^ a bi-directional gated recurrent unit (GRU) module^95^ and a Gumbel tree-GRU module.^4,96^ Each sentence representation *h(s)* is then scored by a sentence-level attention module to indicate its contribution to the relation prediction, that is,

5 $$\begin{array}{*{20}{c}} {\beta \left( s \right) = \frac{{{\mathrm{exp}}\left( {W_s \cdot h\left( s \right)} \right)}}{{\mathop {\sum }\nolimits_{s^{\prime} \in S_{e_1,e_2}} {\mathrm{exp}}\left( {W_s \cdot h\left( {s^{\prime}} \right)} \right)}},} \end{array}$$

where $\beta (s) \in {\Bbb R}$ stands for the weight score, and $W_s \in {\Bbb R}^{d \times 1}$ stands for the learnable weight parameters. Finally, the relation is predicted by a binary classifier, based on the weighted sum of sentence representations, that is,

6 $$\begin{array}{*{20}{c}} {r_{e_1,e_2} = {\mathrm{classifier}}\left[ {\mathop {\sum }\limits_{s \in S_{e_1,e_2}} \beta \left( s \right) \cdot h\left( s \right)} \right],} \end{array}$$

where $r_{e_1,e_2}$ stands for the probability of the relation of interest between entities *e*_1_ and *e*_2_ mentioned by the bag of sentences $S_{e_1,e_2}$.

The training corpus we used was curated automatically from nearly 20 million PubMed (http://www.pubmed.gov) abstracts by a distant supervision technique.^97^ In detail, the names (or aliases) of drugs or targets in sentences were first annotated by a dictionary-based named entity recognition method (string matching), in which the name dictionary was derived from DrugBank (version 5.1.0),^19^ with ambiguous names (e.g., common words) removed. Next, the label for each bag of sentences co-mentioning a drug–target pair of interest was annotated automatically by the known drug–target interactions in DrugBank. The unlabeled corpus that we used in this work for text mining the coronavirus-related drugs was obtained from approximately 2.2 million PMC full-text articles, with entities of interest annotated using the aforementioned named entity recognition approach. A coronavirus-related drug was extracted as a hit candidate if the model found a bag of sentences describing a relation between this drug and a target in the coronavirus of interest.

### Connectivity map analysis

We used the transcriptome analysis approach to further filter the potential drug candidates for treating the COVID-19 patients infected by SARS-CoV-2. We collected the gene expression profiles of the samples from SARS-CoV-2 infected patients to screen the drug candidates against COVID-19. We also used those from SARS-CoV infected patients to filter out the potential therapeutic agents targeting at COVID-19. Such a strategy is reasonable as SARS-CoV and SARS-CoV-2 are two closely related and highly similar coronavirus. First, the genome of SARS-CoV-2 is phylogenetically close to that of SARS-CoV, with about 79% of sequence identity,^98^ and the M (membrane), N (nucleocapsid) and E (envelope) proteins of these two coronaviruses have over 90% sequence similarities.^99^ In addition, the pathogenic mechanisms of SARS-CoV-2 and SARS-CoV are highly similar.^100^

In particular, we collected the gene expression profiles of the peripheral blood mononuclear cells (PBMCs) and the bronchoalveolar lavage fluid (BALF) samples from three SARS-CoV-2 infected patients (NGDC: PRJCA002326)^6^ and PBMC samples from ten SARS-CoV infected patients (GEO: GSE1739).^7^ For samples from SARS-CoV infected patients, the raw gene expression values were first converted into logarithm scale, and then the differential expression values (z-scores) were computed by comparing to those of healthy persons using the same protocol as described in ref. ^5^ that is,

7 $$\begin{array}{*{20}{c}} {Z_{{\mathrm{infected}}} = \frac{{X_{{\mathrm{infected}}} \,-\, {\mathrm{median}}\left( {X_{{\mathrm{healthy}}}} \right)}}{{C \cdot {\mathrm{MAD}}\left( {X_{{\mathrm{healthy}}}} \right)}},} \end{array}$$

8 $$\begin{array}{*{20}{c}} {{\mathrm{MAD}}\left( {X_{{\mathrm{healthy}}}} \right) = {\mathrm{median}}\left( {\left| {X_{{\mathrm{healthy}}} - {\mathrm{median}}\left( {X_{{\mathrm{healthy}}}} \right)} \right|} \right),} \end{array}$$

where *Z*_infected_ stands for the z-scores of the SARS-CoV infected patients, *X*_infected_ and *X*_healthy_ stand for the gene expression values in logarithm scale of the infected and healthy persons, respectively, median(*·*) stands for the median operation, MAD(·) stands for the median absolute deviation operation, and *C* = 1.4826 is a constant for normalization. The *p*-values for all the genes with measured expression values during the analysis were also computed based on the z-scores. For samples from those SARS-CoV-2 infected patients, due to the limited sample sizes (i.e., three samples for the healthy, three PBMC and two BALF samples for the infected patients), using z-score for differentially expressed gene analysis can be inaccurate and misleading. This is because the MAD mentioned above can be of high variance when the number of healthy samples is low (i.e., three), resulting in inaccurate estimation of the z-score. Here we mainly used the fold-change to analyze the differential expressed genes, which was obtained from the original paper.^6^ The up- and downregulated genes were determined by *p*-value <0.01 and sorted according to the z-score/log2-fold-change values. We used the connectivity map (CMap),^5^ which contains the cellular gene expression profiles under the perturbation of 2428 well annotated reference compounds, to measure the associations of gene expression patterns between SARS-CoV-2/SARS-CoV infected patients and the reference compound-perturbed cells. The connectivity map scores were computed based on the up- and downregulated gene sets of SARS-CoV-2/SARS-CoV infected patients using the web tool (https://clue.io/query). Under the hypothesis that the gene expression pattern resulting from the perturbation by a therapeutic compound should be negatively correlated with that resulting from the coronavirus infection, we selected those compounds that have significant negative connectivity map scores, that is, the list of drug candidates predicted to treat the coronavirus infected patients was obtained by selecting the compounds with the connectivity map scores <−90, which was suggested by the original paper.^5^

### Comparing molecular characteristics between PARP1 inhibitors

We compared the molecular characteristics between PJ-34 and other available PARP1 inhibitors that are FDA-approved or in clinical trials, including niraparib, rucaparib, veliparib, nicotinamide, olaparib, iniparib, theophylline, talazoparib, and CVL218. In particular, near 200 molecular characteristics were calculated using RDKit,^88^ by enumerating all the property descriptor functions in rdkit.Chem.Descriptors.descList. These functions calculate the scalar features such as molecular weight, logP and number of heteroatoms. Those functions with the returned invalid values were discarded. As the output molecular features of different functions were not always in comparable scales, each dimension of the molecular characteristics of every PARP1 inhibitor was normalized into range [0,1]. Finally, the Euclidian distance between the normalized feature vectors was used to measure the similarities between two PARP1 inhibitors.

### Cells and virus

The African green monkey kidney Vero E6 cell line was purchased from the Cell Resources Center of Shanghai Institute of Life Science, Chinese Academy of Sciences (Shanghai, China) and cultured in DMEM medium (Gibco Invitrogen, no. 12430-054) containing 10% fetal bovine serum (FBS; Gibco Invitrogen) at 37 °C with 5% CO_2_ atmosphere. BetaCoV/JS03/human/2020 (EPI_ISL_411953), a SARS-CoV-2 virus strain, was isolated from nasopharyngeal swab of a 40-year old female confirmed as COVID-19 case by reverse transcriptase polymerase chain reaction (RT-PCR) in December 2019. The virus was propagated in Vero E6 cells, and the viral titer was determined by the 50% tissue culture infective dose (TCID50) based on microscopic observation of cytopathic effects. All the in vitro SARS-CoV-2 infection experiments were performed in a biosafety level-3 (BLS-3) laboratory in Jiangsu Provincial Center for Diseases Control and Prevention, Jiangsu, China.

### Antiviral drugs

Potential antiviral drugs, including zanamivir, oseltamivir, remdesivir, baricitinib, olaparib, and arbidol, were all provided by MCE (Medchem Express, China). The PARP1 inhibitor mefuparib (CVL218) with a purity of more than 99.0% was provided by Convalife, Shanghai, China.

### Cytotoxicity test and virus infection assay

The cytotoxicity of the tested drugs on Vero E6 cells was determined by the CCK8 assays (Beyotime, China). At 48 h post addition of the tested drugs, 20 μL CCK8 was added to each well and incubated at 37 °C for 1 h. Then optical density was measured at 450 nm. The 50% cytotoxic concentration (CC_50_) values were calculated using GraphPad Prism 5 (GraphPad Software, USA). Vero E6 cells were seeded into 96-well plates with a density of 5 × 104 cells/well for incubation in DMEM medium supplemented with 10% FBS for 16 h in an incubator with 5% CO_2_ at 37 °C, for cells to reach 80% confluent. Then, cell culture medium of each well was removed, and PBS was used to wash the cells once, before evaluating the antiviral efficacy of the tested drugs. Four duplicated wells were made for each dose of drugs, and the cells were pretreated with different doses of antiviral drugs diluted by the cell maintenance solution (50 μL per well) for 1 h. For the virus control and cell control wells, cell medium containing DMSO or only medium of 50 μL per well was added. Next, pretreated or untreated cells in each well were infected with the virus with a multiplicity of infection (MOI) of 0.05 for 2 h. After that, the virus-drug mixture was removed and cells were further cultured with fresh drug-containing medium at 37 °C with 5% CO_2_ atmosphere for 48 h. Then culture supernatant per well was harvested and inactivated at 56 °C for 30 min to further extract and quantify viral RNA. The preliminary in vitro antiviral activities of the tested drugs were first screened at individual concentrations with oseltamivir at 3 μM, zanamivir at 3 μM, baricitinib at 3.2 μM, olaparib at 3.2 μM, arbidol at at 3 μM and 30 μM, and CVL218 at 3 μM and 30 μM, respectively. Then, the concentration-dependent inhibition activity of CVL218 against SARS-CoV-2 replication was performed with 5-fold serial dilutions of the maximum concentration at 50 μM.

### Viral RNA extraction and quantitative real-time PCR (qRT-PCR)

Viral RNA was extracted from culture supernatant using the HP RNA Isolation Kit (Roche) according to the manufacturer’s instructions. RNA was eluted in 30 μL RNase-free water. Reverse transcription was performed with a SARS-CoV-2 nucleic acid detection kit (BioGerm, China) according to the manufacturer’s instructions. The PCR reaction system was configured as follows: 6 μL of qRT-PCR reaction solution, 2 μL of qRT-PCR enzyme mixture, 2 μL of primer probe and 2.5 μL of template, and the reaction was performed as follows: 50 °C for 10 min, 95 °C for 5 min, followed by 40 cycles of 95 °C for 10 s, 55 °C for 40 s. The values of 2^−Δ*CT*^ were calculated according to the CT value measured from the PCR instrument, to represent the relative virus copies of the drug group to the control group. The virus replication inhibition rate (%) was calculated as (1–2^−Δ*CT*^) × 100%. The dose-response curves were plotted according to viral RNA copies and the drug concentrations using GraphPad Prism 5 (GraphPad Software, USA).

For quantification of N gene, total RNA was also extracted from the infected cells using the Rneasy mini kit (Qiagen) according to the manufacturer’s instructions. The viral N gene was quantified by qRT-PCR by using a SARS-CoV-2 nucleic acid detection kit (bioPerfectus technologies, China) according to the manufacturer’s instructions.

### Time-of-addition assay

To facilitate the observation of the antiviral effects of drugs against SARS-CoV-2 at different timing, relative high doses of the tested drugs (CVL218 at 20 μM and remdesivir at 10 μM) were used for the time-of-addition assay. Vero E6 cells with a density of 5 × 10^4^ cells per well were treated with the tested drugs, or DMSO as controls at different stages of virus infection. The cells were infected with virus at an MOI of 0.05. The “Full-time” treatment was to evaluate the maximum antiviral effects, with the tested drugs in the cell culture medium during the whole experiment process, which was consistent with the descriptions in the virus infection assay. For the “Entry” treatment, the tested drug was added to the cells for 1 h before virus infection, and then cells were maintained in the drug-virus mixture for 2 h during the virus infection process. After that, the culture medium containing both virus and the tested drug was replaced with fresh culture medium till the end of the experiment. For the “Post-entry” experiment, virus was first added to the cells to allow infection for 2 h before the virus-containing supernatant was replaced with drug-containing medium until the end of the experiment. At 14 h post infection, the viral inhibition in the cell supernatants of the tested drug was quantified by qRT-PCR, and calculated using the DMSO group as reference.

### Indirect immunofluorescence assay

Vero E6 cells were treated with CVL218 at 5 μM, 15 μM, and 25 μM, respectively, following the same procedure of “full-time” treatment. After 48 h of culture, infected cells were fixed with 80% acetone in PBS and permeabilized with 0.5% Triton X-100, and then blocked with 5% BSA in PBS buffer containing 0.05% Tween 20 at room temperature for 30 min. The cells were further incubated with a rabbit polyclonal antibody against a SARS-CoV nucleocapsid protein (Cambridgebio, USA) as primary antibody at a dilution of 1:200 for 2 h, followed by incubation with the secondary Alexa 488-labeled goat anti-rabbit antibody (Beyotime, China) at a dilution of 1:500. Nuclei were stained with DAPI (Beyotime, China). Immunofluorescence was observed using fluorescence microscopy.

### Western blot assay

NP expression in infected cells was analyzed by western blot. Protein samples were separated by SDS-PAGE and then transferred onto polyvinylidene difluoride membranes (Millipore, USA), before being blocked with 6% Rapid Block Buff II (Sangon Biotech, China) at room temperature for 10 min. The blot was probed with the antibody against the viral nucleocapsid protein (Cambridgebio, USA) and the horseradish peroxidase-conjugated Goat Anti-Rabbit IgG (Abcam, USA) as the primary and the secondary antibodies, respectively. Protein bands were detected by chemiluminescence using an ECL kit (Sangon Biotech, China).

### Inhibitor combination assay

To assess the potential synergistic effect, CVL218 at 3.5 μM and favipiravir at 30 μM were mixed, while CVL218 alone and favipiravir alone were included as controls. The concentrations were selected according to the EC_25_ values of individual drugs against SARS-CoV-2 in vitro. The mixtures were tested for their inhibitory activities on the SARS-CoV-2 with a multiplicity of infection (MOI) of 0.05 as described above. Each sample was tested in triplicate.

### SPR binding assay

Surface plasmon resonance experiments were performed with a BIAcore S200 (GE Healthcare) as previously described.^101^ The running buffer contained 1× PBS, 2% DMSO. The purified SARS-CoV-2-N protein was desalted using Zeba^TM^ spin desalting column (Thermo Scientific), diluted in 10 mM sodium acetate (pH 4.0) to 20 μg/mL, and immobilized on a CM5 sensor chip by amine coupling. The tested drugs in 2-fold serial dilutions were made in the running buffer. All measurements were performed at a flow rate of 30 μL/min. Data processing and analyses were performed using BIAevaluation 1.1 software.

### LPS-induced cytokine production in PBMCs

Peripheral blood mononuclear cells (Allcells) were cultured at 37 °C at concentration 5% CO_2_ atmospheric on a 96-well plate in RPMI1640 cell growth medium (Gibico, Cat. 11875-093). For stimulation, PBMC cells were incubated with 1 μg/mL LPS (Sigma, Cat. L2880-25MG). To test whether CVL218 and olaparib can inhibit the production of IL-6, IL-10, IFN-*γ*, TNF-*α*, 1 μM and 3 μM concentrations of CVL218 or olaparib were added to cell culture medium for 6 and 24 h, respectively. The concentration of each cytokine was determined by ELISA using a commercial kit (BioLegend, USA).

### Pharmacokinetics and toxicity study

Sprague-Dawley rats were purchased from Shanghai Laboratory Animal Center, China. The animals were grouped and housed in wire cages with no more than six per cage, under good laboratory conditions (temperature 25 ± 2 °C; relative humidity 50 ± 20%) and with dark and light cycle (12 h/12 h). Only healthy animals were used for experimental purpose. The pharmacokinetics and biodistribution study in Sprague-Dawley rats was approved by Center for Drug Safety Evaluation and Research, Shanghai Institute of Materia Medica, Chinese Academy of Sciences. A total of 144 Sprague-Dawley rats with each sex were used for toxicity study. Animals were randomly separated into four groups (18/sex/group). CVL218 was administered at doses of 20, 40, 60, and 160 mg/kg. For all the groups, 20 rats (10/sex/group) were randomly selected and euthanized at day 28, and their sections of various tissues and organs were obtained and frozen. Ten (5/sex/group) animals were euthanized after a 28-day drug free period, and their sections of tissues and organs were obtained and frozen. Six (3/sex/group) were euthanized after the blood samples were obtained. For pharmacokinetic and toxicity evaluation, clinical symptoms, mortality and the animals’ body weight were examined. Serum (0.5 mL) was collected to analyze toxicokinetics at different timepoints post drug administration. The plasma concentration-time data were analyzed using a noncompartmental method (Phoenix, version 1.3, USA) to derive the pharmacokinetic parameters.

### Biodistribution study

Thirty Sprague-Dawley rats were randomly divided into three time point groups (3/sex/group). At 3, 6, and 8 h after CVL218 administration, animals were sacrificed, and the brain, heart, lung, liver, spleen, stomach, and kidney tissues were collected. Tissue samples were washed in ice-cold saline, blotted with paper towel to remove excess fluid, and weighed. Tissue samples were fluid, weighted and stored at −20 ± 2 °C until the determination of drug concentration by LC-MS-MS.

### Toxicity study in cynomolgus monkeys

Healthy male and female cynomolgus monkeys aged 3–4 years were purchased from Guangdong Landau Biotechnology, China. The animals were maintained in accordance with the Guide for the Care and Use of Laboratory Animals.

Cynomolgus monkey (5/sex/group) were selected using a computerized randomization procedure, and administered CVL218 by nasogastric feeding at dose levels of 0 (control), 5, 20, 80 mg/kg. Individual dose volumes were adjusted weekly based on body weight of monkeys. The monkeys were observed twice daily for viability/mortality and for any change in behavior, reaction to treatment or ill-health. Electrocardiograms, intraocular pressure, rectal temperature and body weight were recorded. For all the groups, 2/3 of the animals were randomly selected and euthanized at day 28. The remaining animals were euthanized after a 28-day drug free period. Blood samples were taken before and at 0.5, 1, 2, 4, 8, and 24 h post-dose on days 1 and 28 of the treatment period. Pharmacokinetic evaluation was performed using a noncompartmental method (Phoenix, version 1.3, USA) and pharmacokinetic parameters were calculated for individual monkeys.

### Statistical analysis

All data represent the means ± standard deviations (SDs) of n values, where n corresponds to the number of data points used. The figures were prepared using GraphPad Prism 5 (GraphPad Software, USA). The statistical significance was calculated by SPSS (ver.12), and two values were considered significantly different if the *p*-value is <0.05.

## Supplementary information

Supplementary material

## Data Availability

The datasets analysed and the code used in this study are available in the GitHub repository: https://github.com/FangpingWan/CoV-DTI. Source data are provided with this paper.
