# Supplementary material for: An integrative drug repositioning framework discovered a potential therapeutic agent targeting COVID-19
Source: Signal Transduct Target Ther. 2021 Apr 24;6:165. doi: 10.1038/s41392-021-00568-6 (PMC8065335; doi:10.1038/s41392-021-00568-6)
Supplement: Supplementary file 1 — Supplementary material [file 41392_2021_568_MOESM1_ESM.pdf]

Supplementary Materials for

An integrative drug repositioning framework discovered a potential therapeutic agent targeting COVID-19

Yiyue Ge, Tingzhong Tian, Suling Huang, Fangping Wan, Jingxin Li, Shuya Li, Xiaoting Wang, Hui Yang, Lixiang Hong, Nian Wu, Enming Yuan, Yunan Luo, Lili Cheng, Chengliang Hu, Yipin Lei, Hantao Shu, Xiaolong Feng, Ziyuan Jiang, Yunfu Wu, Ying Chi, Xiling Guo, Lunbiao Cui, Liang Xiao, Zeng Li, Chunhao Yang, Zehong Miao, Ligong Chen, Haitao Li, Hainian Zeng, Dan Zhao, Fengcai Zhu, Xiaokun Shen and Jianyang Zeng

Correspondence to: zengjy321@tsinghua.edu.cn

This PDF file includes:

Supplementary Text
Figures S1 to S5
Tables S1 to S8
References

Supplementary Text

The good pharmacokinetic and toxicokinetic characteristics of CVL218 in animals

1. CVL218 has the highest tissue distribution in the lung tissue of rats.

We further performed *in vivo* pharmacokinetic and toxicokinetic evaluation of CVL218 in animals (Methods). We first examined the concentrations of CVL218 over different tissues in rats at different time points post oral administration at different doses (supplementary Fig S4 and supplementary Table S4), which was also previously reported in [1]. Among seven tissues (i.e., lung, spleen, liver, kidney, stomach, heart and brain), we observed that lung had the highest CVL218 concentration, which was 188-fold higher compared to that of plasma (supplementary Table S5). The observation that lung had the highest concentration of CVL218 was in line with the fact that the SARS-CoV-2 virus has the most pathological impact in lung with high viral loads, which suggested that CVL218 has the great potential to be used for the indications of the lung lesions caused by SARS-CoV-2 infection, if its antiviral profile can be established in animal models and clinical trials.

Furthermore, we compared the pharmacokinetic data between CVL218 and arbidol, a broad-spectrum antiviral drug had been recommended to treat the SARS-CoV-2-infected patients [2]. We found that the pharmacokinetic parameters of CVL218 and arbidol were comparable, with similar plasma concentrations and drug exposures (supplementary Table S6). Arbidol was mostly distributed in stomach and plasma post administration in rats (supplementary Table S5). In contrast, higher distributions of CVL218 in tissues especially in lung rather than plasma compared to those of arbidol indicated a superior pharmacokinetic profile of CVL218, which may render it as a better potential antiviral treatment of SARS-CoV-2 infection in lung.

2. The toxicity study demonstrated a safety profile of CVL218 in rats.

In rats after being orally administrated 20/60/160 mg/kg of CVL218 for 28 consecutive days and followed by 28 more days without drug administration (Methods), we observed no significant difference in body weight of rats among different dosage and the control groups (supplementary Fig S5a).

We next conducted a toxicokinetic analysis of CVL218 in rats (Methods). In particular, rats were given CVL218 20/60/160 mg/kg by oral gavage once a day for consecutive 28 days, followed by 28 more days without CVL218 administration, to investigate the reversibility of the toxic effects of the compound and examine whether there is any potential delayed-onset toxicity of this drug in rats. The results showed that, the maximum tolerable dose (MTD) and the no-observed adverse effect level (NOAEL) were 160 mg/kg and 20 mg/kg, respectively. The exposure of female rats to CVL218 (AUC_{0-24}) was 7605 h·ng/mL in day 1 and 6657 h·ng/mL in day 28, while that of male rats (AUC_{0-24}) was 9102 h·ng/mL in day 1 and 10253 h·ng/mL in day 28 (supplementary Table S7). Based on the toxicokinetic results from the repeated dose studies, all rats survived after a 28-day treatment period and showed no apparent sign of toxicity.

3. CVL218 exhibits a favorable safety profile in monkeys.

Monkeys were administered CVL218 (5, 20 or 80 mg/kg) by nasogastric feeding tubes with a consecutive daily dosing schedule for 28 days, followed by a 28-day recovery period (Methods). Only a slight decrease of body weight was observed in the high-dose (80 mg/kg) group, and all changes were reversed after a 28-day recovery period (supplementary Fig S5b), demonstrating a favorable safety profile for CVL218 in monkeys. Further examination of the toxicokinetic data of CVL218 in monkeys showed that the increase of the exposure of CVL218 (AUC_{0-24}) was approximately dose proportional, and after consecutive 28 days of drug administration, the accumulation was not apparent. The exposure of female monkeys to CVL218 (AUC_{0-24}) was 19466 h·ng/ml in day 1 and 18774 h·ng/ml in day 28 (supplementary Table S8), while that of male monkeys (AUC_{0-24}) was 16924 h·ng/ml in day 1 and 22912 h·ng/ml in day 28. The maximum tolerable dose (MTD) of CVL218 in monkeys was 80 mg/kg, and the dose of 5 mg/kg was considered as the no-observed adverse effect level (NOAEL).

Overall, the above *in vivo* data showed that CVL218 possesses good pharmacokinetic and toxicokinetic characteristics in rats and monkeys, and its high-level distribution in the therapeutically targeted tissue (i.e., lung) may greatly favor the treatment of SARS-CoV-2 infection.

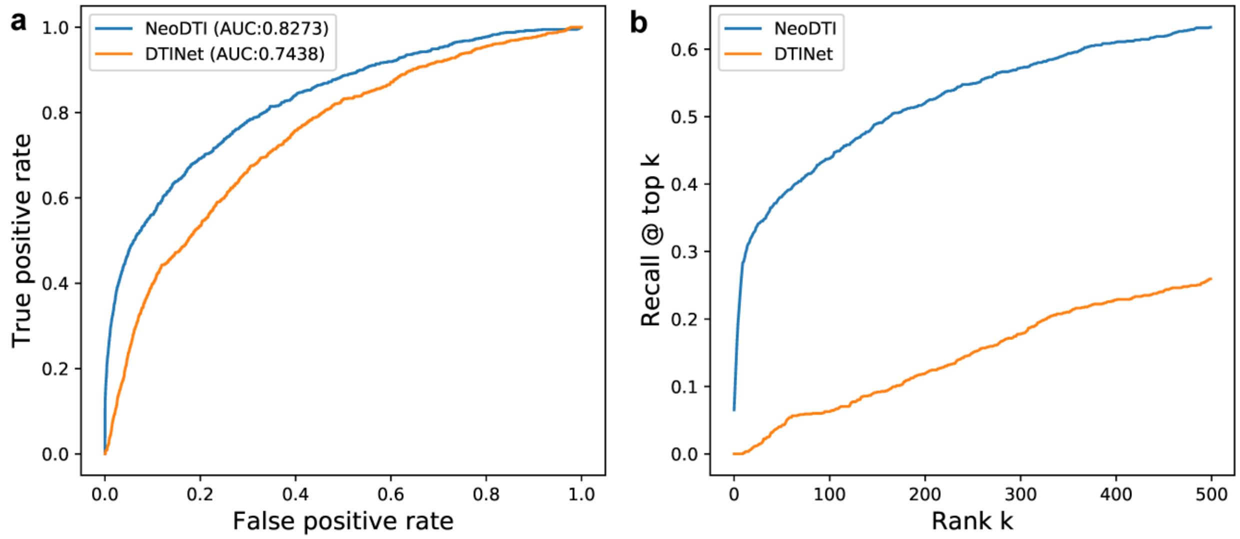

Figure. S1.

Performance evaluation of our network-based knowledge mining algorithm CoV-DTI, using a 10-fold cross-validation procedure. Our previously-developed network-based drug-target interaction (DTI) prediction algorithm DTINet [3] was used as a state-of-the-art baseline for comparison. (a). The receiver operating characteristic (ROC) curves and the corresponding area under the ROC (AUROC) scores. (b). The curves of recall scores with respect to the list of top k drug candidates selected by the prediction algorithms.

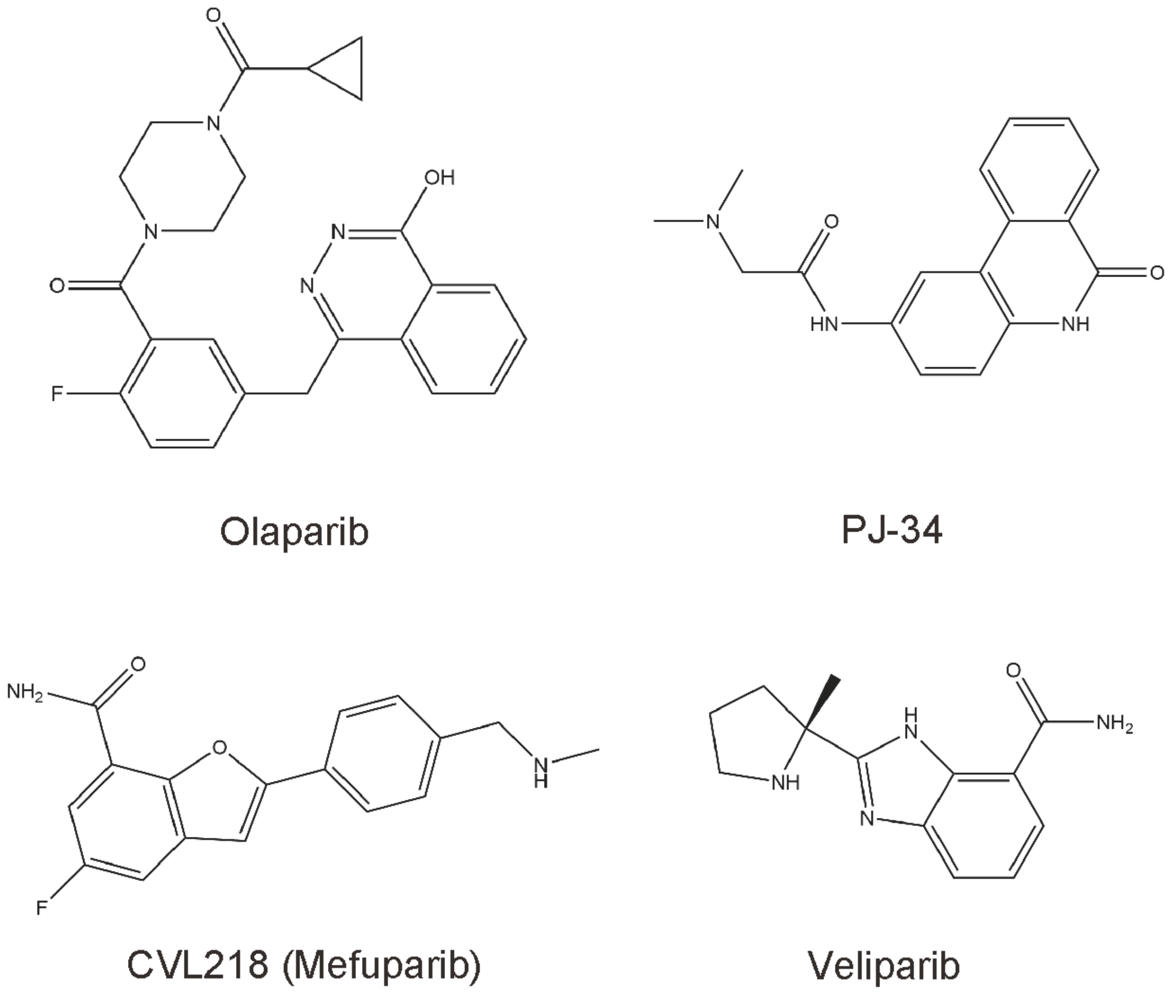

Figure. S2.
Chemical structures of the PARP1 inhibitors mentioned in this study.

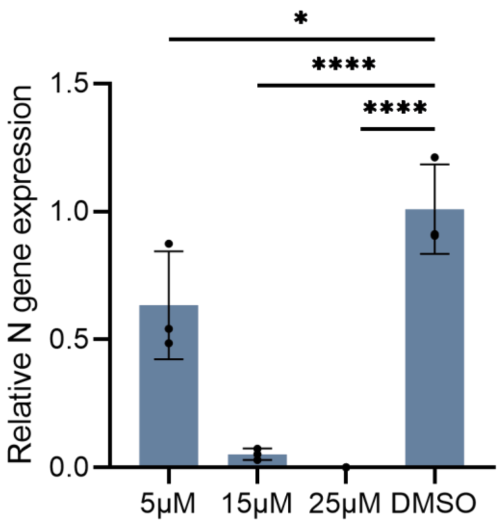

Figure. S3.

Relative N gene expression levels after CVL218 treatment. Relative gene expression levels of nucleoprotein (NP) in the infected Vero E6 cells upon CVL218 treatment (5 μM, 15 μM, 25 μM) and DMSO at 48 h post the SARS-CoV-2 infection were measured using RT-PCR. The results were presented as fold changes relative to control (DMSO) over three independent experiments. Significant levels were calculated according to the one-way ANOVA with Dunnett's multiple comparison tests. ****: $P < 0.0001$, *: $P < 0.05$.

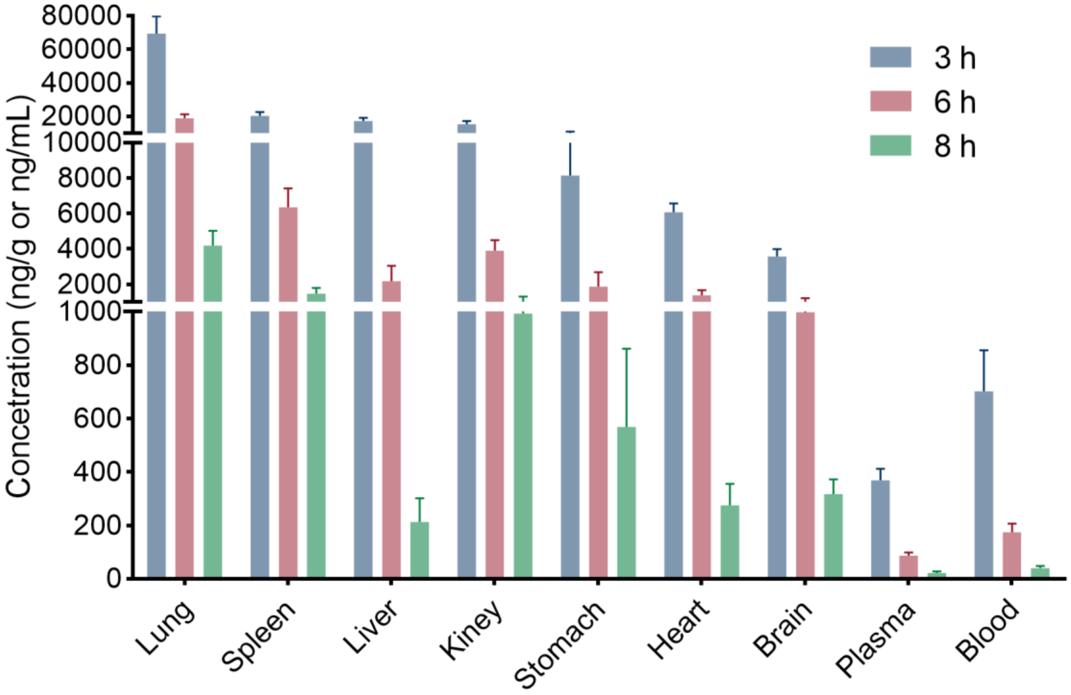

Figure. S4.

Tissue distribution characteristics of CVL218 in rats, with the highest concentration in lung. The concentrations of CVL218 in different tissues were measured at the 3/6/8 h time points after 20 mg/kg oral administration to rats. With the extension of administration time, the concentration of CVL218 in each organ decreased in a time-dependent manner.

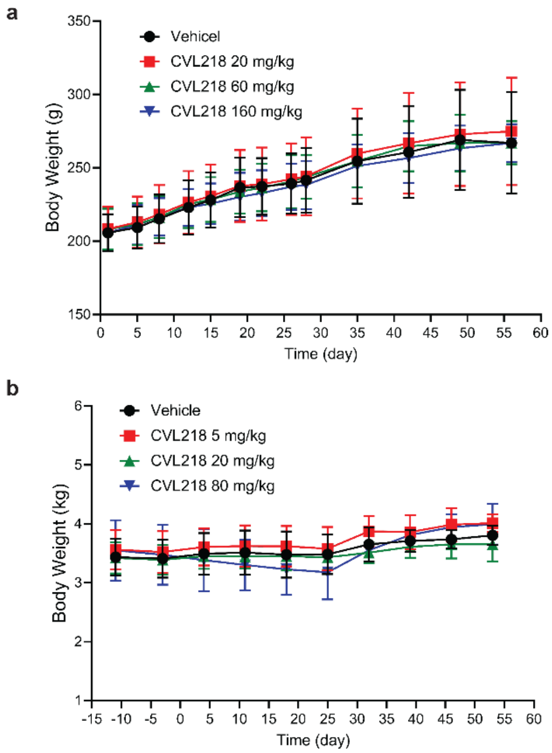

Figure. S5.

Effects of CVL218 on body weight in rats (a) and monkeys (b). Rats and monkeys were orally administered 20/60/160 mg/kg and 5/20/80 mg/kg of CVL218, respectively, for 28 consecutive days and then followed by 28 more days without drug administration. No significant difference was observed between the control group and drug administrated groups at different drug dosages according to two-tailed t-test under the FDR of 0.05.

Rank	DrugBank ID	Drug name
1	DB12695	Phenethyl Isothiocyanate
2	DB11638	Artenimol
3	DB00091	Cyclosporine
4	DB01024	Mycophenolic acid
5	DB00688	Mycophenolate mofetil
6	DB00570	Vinblastine
7	DB00787	Acyclovir
8	DB12480	Betulinic Acid
9	DB00198	Oseltamivir
10	DB04216	Quercetin
11	DB05676	Apremilast
12	DB00795	Sulfasalazine
13	DB00608	Chloroquine
14	DB06246	Exisulind
15	DB00279	Liothyronine
16	DB00277	Theophylline
17	DB07954	3-isobutyl-1-methyl-7H-xanthine
18	DB01229	Paclitaxel
19	DB01248	Docetaxel
20	DB02709	Resveratrol
21	DB00503	Ritonavir
22	DB00244	Mesalazine
23	DB01088	Iloprost
24	DB00945	Acetylsalicylic acid

Rank	DrugBank ID	Drug name
25	DB15588	Ursolic acid
26	DB00541	Vincristine
27	DB08348	PJ-34
28	DB00220	Nelfinavir
29	DB00238	Nevirapine
30	DB00651	Dyphylline
31	DB01179	Podofilox
32	DB01611	Hydroxychloroquine
33	DB01206	Lomustine
34	DB01050	Ibuprofen
35	DB12340	Navitoclax
36	DB05266	Ibudilast
37	DB09221	Polaprezinc
38	DB04115	Berberine
39	DB08912	Dabrafenib
40	DB00877	Sirolimus
41	DB00441	Gemcitabine

Table S1.

The top list of drug candidates identified by CoV-DTI followed by the large-scale relation extraction method BERE and a minimum of manual checking. The drug candidates with both significant prediction scores (p-values < 0.05) and associated with viruses according to text mining results are listed below. Those trivial non-drug candidates (minerals, endogenous substances, chemical reagents and antiseptics) were also excluded from the list.

SARS-CoV-2 (PBMC)			
Connectivity Map Score	Compound BRD ID	Name	Description
−95.34	BRD-K64800655	PHA-793887	CDK inhibitor
−95.03	BRD-K64606589	Apicidin	HDAC inhibitor
−94.57	BRD-K08547377	Irinotecan	Topoisomerase inhibitor
−94.25	BRD-A45498368	WYE-125132	MTOR inhibitor
−93.94	BRD-K28907958	CD-437	Retinoid receptor agonist
−93.55	BRD-K13049116	BMS-754807	IGF-1 inhibitor
−92.98	BRD-K52911425	GDC-0941	PI3K inhibitor
−92.77	BRD-K08502430	Angiogenesis-inhibitor	Angiogenesis inhibitor
−92.54	BRD-K67868012	PI-103	MTOR inhibitor
−91.93	BRD-U33728988	QL-X-138	MTOR inhibitor
−91.90	BRD-K37798499	Etoposide	Topoisomerase inhibitor
−91.78	BRD-K12867552	THM-I-94	HDAC inhibitor
−91.21	BRD-U51951544	ZG-10	JNK inhibitor
−91.21	BRD-K97365803	PI-828	PI3K inhibitor
−90.09	BRD-A80638690	Floxuridine	DNA synthesis inhibitor
−90.02	BRD-M86331534	Pyrvinium-pamoate	AKT inhibitor
SARS-CoV-2 (BALF)			
Connectivity Map Score	Compound BRD ID	Name	Description
−90.51	BRD-K51941867	LM-1685	Cyclooxygenase inhibitor
−90.20	BRD-A51820102	Econazole	Bacterial cell wall synthesis inhibitor
−90.15	BRD-K59184148	SB-216763	Glycogen synthase kinase inhibitor

Table S2.
The remaining drug candidates in the top list identified by the connectivity map analysis using the gene expression profiles of the peripheral blood mononuclear cell (PBMC) samples of three SARS-CoV-2 infected patients and the bronchoalveolar lavage fluid (BALF) samples of two SARS-CoV-2 infected patients [4].

Dataset	AUROC	AUPR
Original knowledge graph	0.9179 ± 0.0024	0.7734 ± 0.0053
Updated knowledge graph	0.9244 ± 0.0026	0.7835 ± 0.0075

Table S3.
Comparison of CoV-DTI on the original knowledge graph and the updated knowledge graph after incorporating the human protein-virus protein interactions derived from [5]. Shown are the mean±SD values over ten replicates of 10-fold cross-validation.

Drugs	Dose (mg/kg)	Time (min)	Lung (ng/g)	Lung (ng/g)	Liver (ng/g)	Kidney (ng/g)	Stomach (ng/g)	Heart (ng/g)	Brain (ng/g)
CVL218	20	180	69318±10476	20202±2300	17215±1919	15201±1984	8145±2624	6077±496	3580±416
		360	18858±2365	6358±1058	2187±859	3903±594	1871±813	1390±292	998±220
		480	4183±847	1475±324	213±88	993±327	569±293	275±80	317±55
Arbidol[a]	54	5	933±837	48±35	104±82	79±54	8210±5410	72±47	101±67
		15	2603±1848	519±281	963±290	259±190	23180±10170	132±69	50±10
		360	833±397	143±51	262±175	58±21	52750±3059	41±28	31±21

a. The concentrations of arbidol in different tissues of rats at 5/15/360 min time points with 54 mg/kg oral administration were obtained from [6].

Table S4.

Comparison of the tissue distributions of CVL218 and arbidol in rats, following 20 mg/kg and 54 mg/kg oral administrations, respectively.

Tissue	Tissue to plasma concentration ratio					
	CVL218			Arbidol		
Lung	188.364	±	28.467	0.553	±	0.392
Spleen	54.897	±	6.250	0.110	±	0.060
Liver	46.780	±	5.215	0.204	±	0.062
Kidney	41.307	±	5.391	0.055	±	0.040
Stomach	22.133	±	7.130	4.920	±	2.159
Heart	16.514	±	1.348	0.028	±	0.015
Brain	9.728	±	1.130	0.011	±	0.002

Table S5.

Comparison of the tissue to plasma concentration ratios between CVL218 and arbidol in rats. The concentrations of CVL218 over different tissues of rats were measured at the 180 min time point following 20 mg/kg oral administration. The concentrations of arbidol over different tissues of rats at the 15 min time point following 54 mg/kg oral administration were obtained from the literature [6, 7]. Means and standard deviations are shown.

Drugs	Dose (mg/kg)	Gender	Tmax (h)	Cmax (ng/mL)	AUC_{0-t} (ng·h/mL)	$AUC_{0-\infty}$ (ng·h/mL)	$MRT_{0-\infty}$ (h)	$t_{1/2}$ (h)
CVL218	20	male	4.0 (4.0$\tilde{4}$.0)	234 ± 35	1070 ± 176	1111 ± 192	3.91 ± 0.19	1.19 ± 0.09
		female	3.0 (2.0$\tilde{3}$.0)	502 ± 80	2196 ± 228	2222 ± 241	3.16 ± 0.41	1.1 ± 0.16
		total	3.5 (2.0$\tilde{4}$.0)	368 ± 157	1633 ± 643	1666 ± 639	3.54 ± 0.50	1.15 ± 0.13
	40	male	3.0 (2.0$\tilde{4}$.0)	510 ± 259	2802 ± 967	2830 ± 983	4.51 ± 0.18	1.3 ± 0.33
		female	2.0 (2.0$\tilde{3}$.0)	940 ± 117	5220 ± 1113	5242 ± 1115	4.05 ± 0.43	1.29 ± 0.21
		total	2.5 (2.0$\tilde{4}$.0)	725 ± 296	4011 ± 1620	4036 ± 1620	4.28 ± 0.39	1.3 ± 0.24
Arbidol[a]	18	male	0.28 ± 0.11	1002 ± 298	1956 ± 895	2224 ± 1058	-	3.6 ± 1.2
	54	male	0.18 ± 0.06	4711 ± 2361	6790 ± 2749	7558 ± 2877	-	3.3 ± 0.7

a. The pharmacokinetic data of arbidol were obtained from [7].

Table S6.

Comparisons of the pharmacokinetic parameters in rats between CVL218 and arbidol following 20/40 mg/kg and 18/54 mg/kg oral administrations, respectively.

				Toxicological parameters					
				Day 1			Day 28		
Group	Gender	Dose (mg/kg)		T_{max} (h)	C_{max} (ng/mL)	AUC_{0-24} (h·ng/mL)	T_{max} (h)	C_{max} (ng/mL)	AUC_{0-24} (h·ng/mL)
1	M	20	Mean	3.00	261	2373	3.00	147	1004
			SD	0.00	124	2000	0.00	61.0	431
			N	3	3	3	3	3	3
	F	20	Mean	3.00	314	1674	3.00	147	797
			SD	0.00	56.2	382	0.00	35.7	197
			N	3	3	3	3	3	3
2	M	60	Mean	5.00	513	6784	3.00	611	5610
			SD	0.00	119	1592	0.00	114	1343
			N	3	3	3	3	3	3
	F	60	Mean	5.30	708	9092	2.30	453	4090
			SD	2.50	137	549	1.20	115	312
			N	3	3	3	3	3	3
3	M	160	Mean	6.00	659	9102	5.30	824	10253
			SD	1.70	77.1	1776	2.50	268	3008
			N	3	3	3	3	3	3
	F	160	Mean	2.30	614	7605	3.00	629	6657
			SD	1.20	122	1056	2.00	213	4592
			N	3	3	3	3	3	3

Table S7.
Toxicokinetic parameters of CVL218 in rats in a four-week toxicity study.

				Toxicological parameters					
				Day 1			Day 28		
Group	Gender	Dose (mg/kg)		T_{max} (h)	C_{max} (ng/mL)	AUC_{0-24} (h·ng/mL)	T_{max} (h)	C_{max} (ng/mL)	AUC_{0-24} (h·ng/mL)
1	M	5	Mean	2.2	119	528	2.8	48	215
			SD	0.4	31.7	138.2	1.3	12.5	62.3
			N	5	5	5	5	5	5
	F	5	Mean	4	76	451	3.8	36	172
			SD	1.4	38.8	239.8	1.6	20.3	77.7
			N	5	5	5	5	5	5
2	M	20	Mean	4	440	4838	5	239	2111
			SD	2.5	162.4	2086.4	0.0	91.1	1186.8
			N	5	5	5	5	5	5
	F	20	Mean	4.6	479	4963	4.6	322	2779
			SD	0.9	100.5	1189.5	0.9	125.4	1458
			N	5	5	5	5	5	5
3	M	80	Mean	3.4	1372	16924	6.8	1582	22912
			SD	0.9	617.4	8831.1	1.6	416.6	8859.6
			N	5	5	5	5	5	5
	F	80	Mean	5.2	1389	19466	5.6	1403	18774
			SD	1.8	387.5	5535.4	2.5	489.6	6179.1
			N	5	5	5	5	5	5

Table S8.

Toxicokinetic parameters of CVL218 in monkeys in a four-week toxicity study.

References.

[1] J.-X. He, M. Wang, X.-J. Huan, C.-H. Chen, S.-S. Song, Y.-Q. Wang, X.-M. Liao, C. Tan, Q. He, L.-J. Tong, et al., Novel PARP1/2 inhibitor mefuparib hydrochloride elicits potent in vitro and in vivo anticancer activity, characteristic of high tissue distribution, Oncotarget 8 (3) (2017) 4156.

[2] C. Chen, J. Huang, Z. Cheng, J. Wu, S. Chen, Y. Zhang, B. Chen, M. Lu, Y. Luo, J. Zhang, et al., Favipiravir versus arbidol for COVID-19: A randomized clinical trial, medRxiv.

[3] Y. Luo, X. Zhao, J. Zhou, J. Yang, Y. Zhang, W. Kuang, J. Peng, L. Chen, J. Zeng, A network integration approach for drug-target interaction prediction and computational drug repositioning from heterogeneous information, Nature communications 8 (1) (2017) 1–13.

[4] Y. Xiong, Y. Liu, L. Cao, D. Wang, M. Guo, D. Guo, W. Hu, J. Yang, Z. Tang, Q. Zhang, et al., Transcriptomic characteristics of bronchoalveolar lavage fluid and peripheral blood mononuclear cells in COVID-19 patients, Available at SSRN 3549993.

[5] D. E. Gordon, G. M. Jang, M. Bouhaddou, J. Xu, K. Obernier, K. M. White, M. J. O'Meara, V. V. Rezelj, J. Z. Guo, D. L. Swaney, et al., A SARS-CoV-2 protein interaction map reveals targets for drug repurposing, Nature (2020) 1–13.

[6] X. Liu, K. Pei, X.-h. Chen, K.-s. Bi, Distribution and excretion of arbidol hydrochloride in rats, Chinese journal of new drugs 22 (7) (2013) 829–833.

[7] X. Liu, Q.-g. Zhou, H. Li, B.-c. Cai, X.-h. Chen, K.-s. Bi, Pharmacokinetics of arbidol hydrochloride in rats, Chinese pharmacological bulletin 28 (12) (2012) 1747–1750.
